# Microbiome modulation of tumorigenesis and immune responses

**DOI:** 10.1186/s12929-025-01208-9

**Published:** 2026-01-05

**Authors:** Yuning Chen, Ying Fang, Zibai Lyu, Yanxin Tian, Siyue Niu, Yan-Ruide Li, Lili Yang

**Affiliations:** 1 Department of Microbiology, Immunology & Molecular Genetics, University of California, Los Angeles, Los Angeles, CA 90095 USA; 2 Department of Bioengineering, University of California, Los Angeles, Los Angeles, CA 90095 USA; 3 Molecular Biology Institute, University of California, Los Angeles, CA 90095 USA; 4 Eli and Edythe Broad Center of Regenerative Medicine and Stem Cell Research, University of California, Los Angeles, Los Angeles, CA 90095 USA; 5 Jonsson Comprehensive Cancer Center, David Geffen School of Medicine, University of California, Los Angeles, Los Angeles, CA 90095 USA; 6 Parker Institute for Cancer Immunotherapy, University of California, Los Angeles, Los Angeles, CA 90095 USA

**Keywords:** Microbiome, Microbial metabolites, Tumorigenesis, Immune modulation, Cancer immunotherapy, Dysbiosis, Microbiome-targeted therapies

## Abstract

The microbiome has emerged as a critical, context-dependent regulator of tumorigenesis and anticancer immunity, capable of either promoting cancer progression or protecting against malignancy. This dual role is mediated by multiple interconnected mechanisms—including chronic inflammation, modulation of immune responses, and alterations in host metabolic signaling. These microbiome-cancer interactions vary across organs, influencing malignancies in the colon, breast, lung, and beyond. Clinically, the microbiome significantly affects patient responses to cancer therapies, particularly immunotherapies such as immune checkpoint blockade (ICB) and chimeric antigen receptor (CAR)-T cell therapy. Although emerging therapeutic strategies aimed at modulating the microbiome have shown promising early results, challenges remain, including individual microbiome variability and the dynamic interplay between the immune system and microbial communities. Nevertheless, harnessing the microbiome holds significant potential to transform precision oncology, offering personalized cancer prevention and treatment strategies tailored to each patient’s unique microbial ecosystem.

## Introduction

Over the past decade, it has become increasingly clear that the commensal microbiome plays a pivotal and context-dependent role in cancer biology. The microorganisms inhabiting barrier sites can profoundly influence tumor initiation, progression, and the host immune response to cancer. Also, disruptions in the normal microbial community, dysbiosis, are now frequently associated with malignancy and have been proposed as a new hallmark of cancer​ [1, 2]. On the other hand, the microbiome can exert protective effects and maintain immune surveillance in some contexts, underscoring the dualistic, context-dependent nature of host-microbiome interactions in cancer. An increasing number of studies underscore that the net effect of the microbiome on cancer is highly situation-specific: while some microbial communities promote inflammation and immune evasion that favor tumor growth, others can stimulate anti-tumor immunity or produce metabolites that restrain neoplastic development​ [2, 3]. As a result, the microbiome has emerged as a crucial extrinsic factor influencing both cancer risk and therapy outcomes across a diverse range of malignancies, from gastrointestinal to lung, breast, and beyond.

Crucially, the microbiome not only influences whether tumors form and grow, but also how the immune system recognizes and combats established cancers. The immune system is deeply intertwined with the microbiome from early development, as commensal microbes continually calibrate immune cell maturation and function​ [1, 4]. In some cases, microbial dysbiosis dampens anti-tumor immunity as seen with *Fusobacterium nucleatum*-mediated expansion of M2-like tumor-associated macrophages and myeloid-derived suppressor cells that inhibit cytotoxic T cell responses in the tumor microenvironment​ [5–8]. In contrast, commensal microbiome can augment anti-cancer immunity. For example, certain microbes can activate dendritic cells, improve T cell infiltration into tumors, and produce metabolites that reinvigorate exhausted T cells and counteract the immunosuppressive tumor microenvironment (TME) [3, 9–13]. Thus, depending on its composition, the microbiome can either impede immune-mediated tumor clearance or facilitate it, which in turn influences how well patients respond to immunotherapies.

The clinical implications of microbiome-cancer interactions are profound. Multiple clinical studies have exhibited that responders to immune checkpoint blockade (ICB) often have distinct gut microbiome profiles compared to non-responders​ [3, 14–17]. Hence, there is growing interest in microbiome-targeted interventions as adjuncts to cancer treatment. Approaches such as fecal microbiota transplantation (FMT), dietary modulation of fiber and nutrient content, and administration of probiotics or consortium of beneficial bacteria, are being actively explored to favorably shift a patient’s microbiome​ [1, 18–20]. While such interventions are still experimental, they underscore a new paradigm of cancer therapy that extends beyond targeting the tumor itself to managing the patient’s microbial ecosystem. In summary, the microbiome has emerged as a potent regulator of tumorigenesis and anticancer immunity, and a deeper understanding of how microbial communities and their metabolites interfere with the immune system holds considerable promise for improving cancer therapy outcomes.

## Microbiota-driven tumorigenesis: organ-specific associations and dual mechanisms

**Fig. 1:**
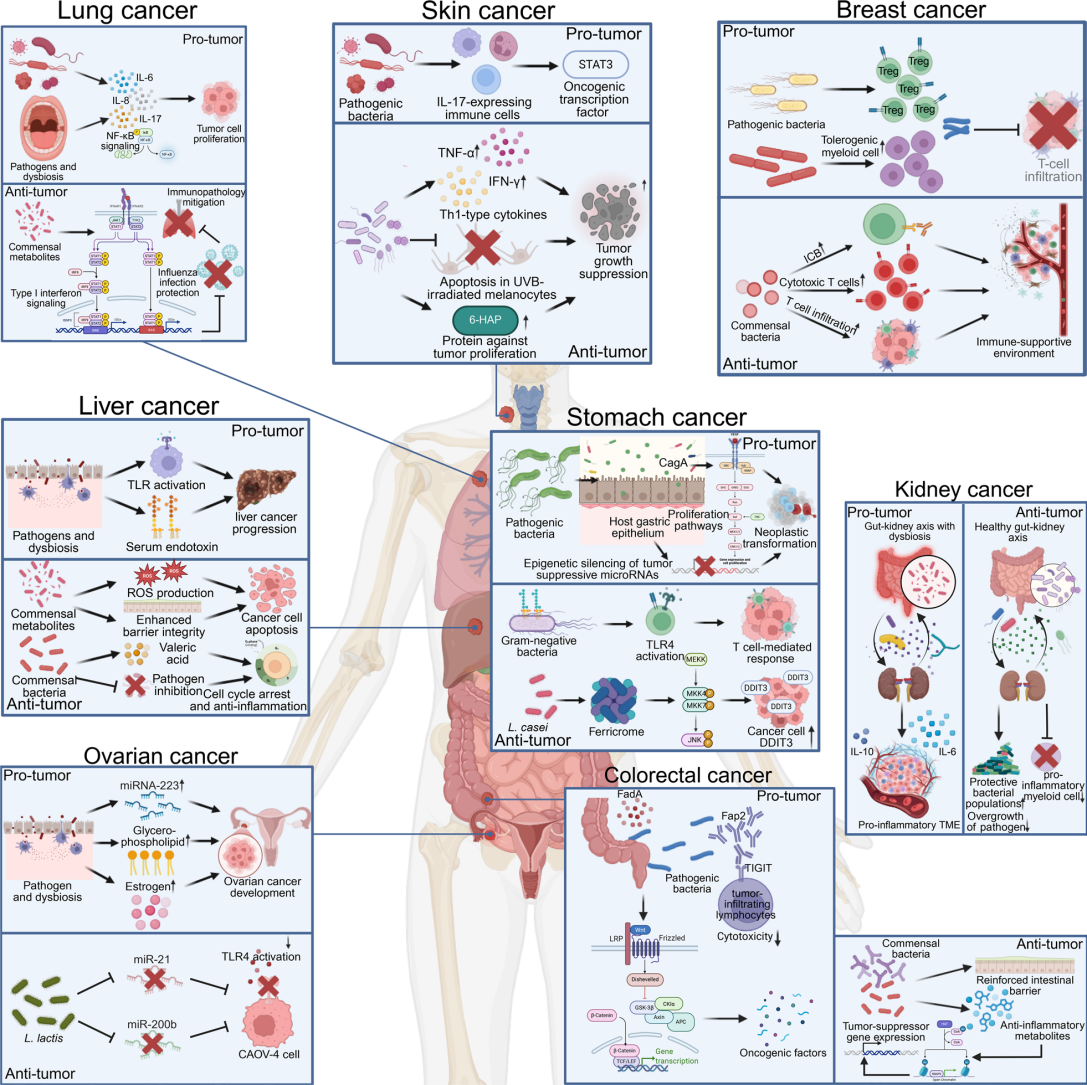
Organ-specific microbiota associations with tumorigenesis. This figure illustrates the associations between specific microbiota and tumorigenesis in various human organs. It highlights the presence of distinct microbial communities within tumor tissues, which are often tumor type-specific and may directly influence cancer development and progression. The figure also depicts how certain bacteria can contribute to carcinogenesis through mechanisms such as the release of toxins causing organ damage, modulation of the immune microenvironment, and induction of chronic inflammation. Additionally, it emphasizes the role of gut microbiota in regulating tumor-promoting and tumor-suppressing pathways associated with inflammation and immunity. ​

The human microbiota exerts a profound influence on tumorigenesis, with complex, context-dependent roles that can either promote or suppress cancer development (Fig. 1) [21]. This duality arises from the intricate interactions between host-associated microbial communities and key cellular pathways governing inflammation, immune surveillance, and metabolism. On one hand, certain microbial species facilitate tumor progression by inducing chronic inflammation, generating genotoxic metabolites, and modulating immune evasion mechanisms [22, 23]. Conversely, commensal and probiotic bacteria contribute to tumor suppression by maintaining epithelial integrity, modulating immune homeostasis, and producing bioactive metabolites such as short-chain fatty acids (SCFAs), which exert anti-inflammatory and anti-tumorigenic effects [24, 25]. Given these dynamic and tissue-specific influences, understanding the organ-specific associations of microbiota with tumorigenesis is crucial for developing and improving microbiota-targeted cancer immunotherapies.

### Breast cancer

Microbial communities in both the gut and breast tissue interact with the immune system in ways that can either promote or inhibit tumorigenesis. Recent tissue-level studies confirm a distinct intratumoral breast microbiome, with compositional differences between malignant and non-malignant breast tissue, supporting a local microbe–tumor interface in breast cancer [26, 27]. Alterations in microbiome composition have been linked to immune evasion by breast tumors, as certain bacterial species facilitate the expansion of regulatory T cells (Treg) and tolerogenic myeloid cells, thereby suppressing cytotoxic immune responses [28]. Additionally, dysbiosis, or microbial imbalance, in the gut and breast microbiome plays a crucial role in breast cancer by influencing immune response and hormone metabolism [29]. Although the origin of microbes in breast cancer remains unknown, increased levels of pathogenic bacteria such as *Escherichia coli*, *Propionibacterium jensenii*, and *Methylobacterium radiotolerans* have been observed in malignant breast tissues compared to healthy controls​, and *Brevundimonas diminuta* and *Peptoniphilus indolicus* have been related to triple-negative breast cancer (TNBC) [30, 31]. Moreover, bacteria like *F. nucleatum* can translocate to breast cancer tissues and hamper T-cell infiltration, facilitating tumor progression and metastasis [32, 33]. Beyond immune exclusion, *F. nucleatum* promotes breast-cancer growth and metastasis via TLR4-dependent pathways and small extracellular vesicles, providing a concrete adhesion-innate-sensing mechanism in the breast tumor microenvironment (TME) [34, 35]. Conversely, certain microbes enhance anti-tumor immunity by promoting the recruitment and activation of cytotoxic T cells. For example, microbial metabolites like indole-3-propionic acid have been shown to boost T cell infiltration and sensitize immune checkpoint blockade treatment in breast cancer models [36]. Furthermore, commensal bacteria residing in breast tissue, particularly *Lactobacillus* species, may might contribute to an immune-supportive environment, as suggested by studies noting that healthy breast tissue is enriched in *Lactobacillus* compared to tumor tissue which harbors more potential pathogens [30, 37, 38].

Beyond immune modulation, the microbiota plays a key role in estrogen metabolism, which is particularly relevant in hormone-dependent breast cancers [39–41]. By modulating estrobolome, a set of bacterial genes encoding β-glucuronidase enzymes that deconjugate and reactivate estrogens, certain bacteria, including *Collinsella* and *Bifidobacterium*, can regulate the bioavailability of estrogen, allowing its reabsorption into circulation rather than excreted [28, 30, 42–44]​. Studies have observed that elevated endogenous estrogen levels have been associated with a higher risk of estrogen receptor-positive breast cancer [28, 45–47]. On the other hand, a balanced microbiome may help regulate estrogen levels by promoting estrogen excretion rather than reabsorption. For instance, lactic acid bacteria such as *Lactobacillus* can suppress β-glucuronidase activity, thereby limiting the deconjugation and reactivation of intestinal estrogens [48]​. Although studies have shown mixed results on whether fiber ultimately lowers systemic estrogen​, high-fiber diets that reduce some *Clostridium* species which enhance β-glucuronidase activity have been associated with lower systemic estrogen levels and potential reduced breast cancer risk [28, 49, 50].

Overall, the interplay between microbiota and breast cancer represents a complex and dynamic relationship. While dysbiosis can contribute to breast cancer progression through promoting immune evasion and increasing estrogen bioavailability, certain microbial communities exert protective effects by enhancing immune responses and regulating metabolic pathways [29].

### Colorectal cancer

Gut microbiota can profoundly influence colorectal cancer (CRC) development. Dysbiosis, an imbalance in the gut microbial community, is often observed in CRC patients, typically marked by an overrepresentation of certain pro-tumorigenic bacteria and a reduction in beneficial species [51]​. One of the most prominently enriched bacteria in CRC tissues is *F. nucleatum*, an oral anaerobe linked to tumor progression [52]. By adhering E-cadherin on the surface of colon cells via FadA adhesin, *F. nucleatum* activates Wnt/β-catenin signaling and induces the expression of oncogenic factors [53]. The adhesin Fap2 of *F. nucleatum* binds to the inhibitory immune receptor TIGIT, suppressing the cytotoxic activity of tumor-infiltrating lymphocytes [54]. *F. nucleatum* also creates a pro-inflammatory microenvironment. Its surface lipopolysaccharide and other virulence factors activate toll-like receptors (TLR) and inflammatory pathways in colon cells, such as inducing microRNA-21 via TLR4, resulting in chronic mucosal inflammation that supports tumor growth [55]​. Clinically, high intratumoral *F.* *nucleatum* has been associated with more aggressive disease, including lower relapse-free survival and even chemoresistance to 5-fluorouracil, as this bacterium can inhibit cancer cell apoptosis under treatment [51, 56]. These findings underscore *F.* *nucleatum* as a pro-tumor microbe and potential therapeutic target in CRC. Beyond FadA-E-cadherin signaling, *F. nucleatum* displays tumor tropism via its Fap2 lectin binding to Gal-GalNAc glycans enriched on CRC cells, facilitating intratumoral enrichment and immune evasion [57–59]. Fap2 concurrently ligates TIGIT on NK and CD8⁺ T cells to blunt cytotoxicity, reinforcing local immune suppression [59, 60]. Together, these adhesion-checkpoint interactions couple microbial colonization to impaired antitumor effector function.

Besides *Fusobacterium*, other gut bacteria produce virulence factors that drive CRC. Certain strains of *E. coli* produce colibactin that can cause DNA cross-links and double-strand breaks in colonic epithelial cells​, leading to mutations and genomic instability that initiate tumorigenesis, while *Bacteroides fragilis* toxin damages the colonic epithelial barrier and activates pro-carcinogenic signaling pathways [54, 61]. Moreover, *Salmonella* produces AvrA, a protein that can activate β-catenin/STAT3 signaling in the colon, enhancing tumorigenesis [54]. Overall, tumor-promoting bacteria leverage mechanisms such as genotoxicity, inflammation, and immune modulation to foster an environment conducive to colorectal cancer.

In contrast, many commensal bacteria exert anti-tumorigenic effects in the colon. Health-associated gut microbes help maintain epithelial homeostasis, prevent chronic inflammation, and even directly inhibit tumor cells. Notably, beneficial genera like *Bifidobacterium* and *Lactobacillus* are often found at lower levels in CRC patients, suggesting their loss may remove a protective influence [62]. In CRC, depletion of butyrate-producing taxa such as *Faecalibacterium prausnitzii* and *Roseburia spp.* is recurrently observed, consistent with loss of SCFA-mediated epithelial and immune regulation [63, 64]. These bacteria are known for reinforcing the intestinal barrier, producing anti-inflammatory metabolites, and outcompeting pathogenic species. In experimental models, supplementation with probiotic strains has demonstrated protective effects against CRC. For example, administering *Lactobacillus* or *Bifidobacterium* strains in models like Azoxymethane (AOM)/Dextran sodium sulfate (DSS) mice reduced the development of tumors [51].

A key feature of protective gut microbes is their capacity to produce the microbial metabolite SCFAs, especially butyrate, through fermentation of dietary fiber [65]. These metabolites generally have tumor-suppressive properties. For example, butyrate possesses anti-inflammatory and anti-tumor effects by serving as a histone deacetylases inhibitor in colon cells​. This epigenetic modulation can maintain histone hyperacetylation, thereby reactivating tumor-suppressor genes and induce growth arrest [66, 67]. While butyric acid protects normal cells by supporting oxidative processes and promoting proliferation, it has the opposite effect on neoplastic cells by inducing apoptosis, possibly related to the inhibition of the Wnt/β-catenin pathway [68, 69]. This suppression is dose-dependent, with cellular suppression increasing progressively at higher butyrate concentrations [68]. In cellular and animal models, adequate butyrate production has been linked to protection against CRC​ [54].

### Kidney cancer

Emerging research suggests a connection between the gut microbiome and kidney cancer, particularly clear cell renal cell carcinoma (ccRCC), despite the resident microbiome of kidney tumors being relatively unexplored. In ccRCC, loss of VHL and consequent stabilization of HIF-2α anchors tumor metabolism, angiogenesis, and inflammatory tone, providing a plausible interface through which gut-derived metabolites and cytokines can shape renal oncogenesis and therapy response [70]. The etiology of ccRCC remains unclear, but metabolism and inflammation are implicated in its progression [71]. A proposed gut-kidney axis, mediated by metabolic and inflammatory processes, may influence renal physiology and pathology through alterations in gut microbiota composition. Several findings support the existence of this gut-kidney axis in kidney cancer. For instance, changes in gut microbiota characteristics have been observed in individuals with renal-related diseases, with metabolic status playing a key role in disease progression [71]. Mechanistically, dysbiosis increases gut-derived uremic toxins that amplify oxidative stress and systemic inflammation, lesions that can aggravate kidney injury and create a pro-tumor environment [72]. Altered gut microbiota can lead to the long-term production of uremic toxins, contributing to abnormal renal function [73]. Additionally, gut dysbiosis and the subsequent leakage of pro-inflammatory molecules can establish a chronic inflammatory state, potentially exacerbating kidney diseases. Pro-inflammatory factors such as IL-6, IL-10, and IL-1β, along with estrogen, have been linked to the pathogenesis of ccRCC [74–77]. Increased expression of IL-8 and its receptor CXCR1 has also been associated with ccRCC tumorigenesis [78].

Further evidence suggests that microbial composition differs between ccRCC patients and healthy individuals, suggesting that specific gut microbiota components may contribute to tumorigenesis. Notably, *Desulfovibrionaceae*, a family of sulfide-degrading bacteria, is enriched in the ccRCC group, whereas *Lactobacillus* species appear less abundant in individuals with RCC [71]. *Lactobacillus* and other commensal bacteria not only compete with pathogenic microbes for space and nutrients but also help maintain a stable and beneficial microbial environment [79]. This ecological cooperation supports the expansion of protective bacterial populations, which can suppress the overgrowth of opportunistic pathogens and sustain intestinal health even under inflammatory conditions [79]. Other studies also highlight the impact of antibiotic use on therapy efficacy in malignant kidney tumors. Disruption of the gut microbiome due to uncontrolled antibiotic use may influence oncological disease progression [80]. In patients with advanced RCC undergoing targeted therapy and immunotherapy, antibiotic therapy has been linked to decreased objective response rates to immune checkpoint inhibitors, suggesting a negative impact on treatment outcomes [81].

The metabolic activity of gut microbiota is also considered a key factor affecting renal disease progression. Uremic toxins derived from dysbiotic gut microbiota contribute to renal dysfunction [73]. Metabolomics analyses have identified differences in serum metabolites between ccRCC patients and healthy controls, including a reduction in taurine levels and alterations in arachidonic acid metabolism, which coincided with an increased presence of sulfide-degrading bacteria [71]. Additionally, SCFAs produced by gut microbiota have been shown to mitigate oxidative stress-induced kidney damage [73]. Beyond renal protection, SCFAs, including butyrate, acetate, and propionate, possess anti-tumor properties by suppressing pro-inflammatory myeloid cell activity, promoting goblet cell-derived mucus secretion to fortify the intestinal barrier, and curbing epithelial proliferation in tumor-prone settings. Butyrate, in particular, acts as a histone deacetylase inhibitor, dampening cytokine expression and limiting the transcription of genes involved in oncogenesis [82–84]. Correlations between gut microbiota composition and clinical indices of ccRCC further support the notion that microbial metabolites may influence disease characteristics.

### Liver cancer

The gut-liver axis plays a critical role in the development of hepatocellular carcinoma (HCC) through a complex bidirectional relationship involving metabolic exchange and immunoregulation [85]. Mechanistically, a circuit of bile acid, CXCL16, CXCR6, and NKT cell links gut microbes to hepatic anti-tumor surveillance: microbial 7α-dehydroxylation of bile acids lowers sinusoidal CXCL16, reduces CXCR6⁺ NKT cell accumulation, and favors hepatocarcinogenesis. Conversely, restoring beneficial taxa or bile-acid composition increases CXCL16 and restrains HCC [86]. The liver receives over 70% of its blood supply from the portal vein, exposing it to a range of gut microbiome (GM)-derived molecules, including microbe-associated molecular patterns (MAMPs) and metabolites, which contribute to hepatocarcinogenesis [87]. Dysbiosis of the gut microbiome has been implicated in the progression of chronic liver diseases (CLDs) such as non-alcoholic fatty liver disease (NAFLD) and cirrhosis, increasing the risk of HCC. Distinct microbiome signatures have been identified in HCC patients, underscoring the potential of microbial markers for early diagnosis [88].

Several mechanisms mediate the influence of the gut microbiome on HCC development. Intestinal barrier dysfunction caused by inflammation and dysbiosis allows the translocation of microorganisms and MAMPs into the liver. These microbial components, including pathogen-associated molecular patterns (PAMPs), activate toll-like receptors (TLRs), triggering inflammatory responses that promote tumorigenesis. Elevated serum endotoxin levels, such as lipopolysaccharide (LPS), have been observed in cirrhotic and HCC patients, further supporting the role of gut-derived inflammation in liver cancer progression [89, 90].

Microbial metabolites also play a crucial role in modulating liver homeostasis and influencing HCC development. Short-chain fatty acids (SCFAs), such as butyrate, exert anti-inflammatory and anticancer effects by enhancing barrier integrity and inducing tumor cell apoptosis via reactive oxygen species (ROS) generation [91]. However, some studies suggest that SCFAs may have concentration-dependent effects, with high levels potentially promoting HCC progression [92]. Bile acids (BAs) also contribute to liver tumorigenesis, with an imbalance between primary and secondary BAs influencing cancer risk. For instance, deoxycholic acid (DCA), a secondary BA, has been associated with oncogenic signaling and cellular senescence, whereas other BAs are involved in immunosurveillance of liver tumors [93].

Specific bacterial species have been linked to HCC pathogenesis. *Klebsiella pneumoniae* is enriched in HCC patients and has been implicated in oncogenic pathway activation following hepatic translocation [90]. An overabundance of *E. coli* has been observed in cirrhotic patients with HCC, suggesting its role in malignant transformation. Additional shifts in bacterial composition, including increased *Enterococcus*, *Streptococcus*, *Akkermansia*, and *Bifidobacterium*, as well as decreased *Blautia* and *Agathobacter* (SCFA-producing bacteria), have been identified in NAFLD-related HCC [94–97]. Dysbiosis can also affect liver immunity, as seen with *Odoribacteraceae*, which produces *isoallolithocholic* acid (isoallo-LCA), a metabolite that induces immunosuppressive liver-resident macrophages. Disruptions in this regulatory system may contribute to HCC progression [98]. The inflammatory milieu created by gut dysbiosis further promotes HCC by activating pro-inflammatory signaling pathways and increasing cytokine production [98]. Persistent inflammation facilitates tumor initiation and progression by promoting hepatocyte damage and proliferation.

Emerging evidence suggests that *Lactobacillus* species may exert protective effects against liver disease and HCC. While its role in liver cancer remains under investigation, *Lactobacillus* has demonstrated anti-tumor properties in colorectal cancer models, highlighting its potential influence on the gut-liver axis. In preclinical studies, treatment with a commercial probiotic formulation VSL#3 from VSL Pharmaceutical, containing *Lactobacillus* species, restored gut dysbiosis, improved mucosal barrier integrity, reduced serum LPS levels, and inhibited HCC development in a chemically induced rat model of HCC [90]. Complementing this, Lactobacillus acidophilus suppresses NAFLD-HCC via the postbiotic valeric acid, which binds GPR41/43 on hepatocytes to inhibit Rho-GTPase signaling, reduce tumor formation, and improve intestinal barrier integrity in multiple mouse models [99]. Similarly, *Lactobacillus rhamnosus GG* culture supernatant has been shown to mitigate acute alcohol-induced intestinal permeability and liver injury in mice, suggesting its protective role against liver damage, a known risk factor for HCC [100]. Oral supplementation with *Lactobacillus acidophilus* in NAFLD-HCC animal models has demonstrated antitumor effects through the production of valeric acid, which suppresses the Rho-GTPase pathway and induces cell cycle arrest in hepatic cells [101]. In contrast, studies on intrahepatic cholangiocarcinoma have reported higher levels of *Lactobacillus* in patients compared to HCC and control groups, indicating potentially distinct roles of *Lactobacillus* in different hepatobiliary cancers. In addition to its direct effects on tumor cells, *Lactobacillus* contributes to gut homeostasis by inhibiting the colonization and overgrowth of pathogenic bacteria, thereby reducing infection-associated inflammatory burdens on the liver. *Lactobacillus brevis CD2* lozenges have demonstrated efficacy in reducing radiation- and chemotherapy-induced mucositis in head and neck cancer patients, suggesting a potential role in mitigating treatment-related toxicities [102]. Furthermore, *Lactobacillus casei* strains that overproduce linoleic acid have been shown to limit the growth and virulence of *Salmonella Typhimurium* and enterohaemorrhagic *E. coli*, further supporting the probiotic’s role in maintaining gut-liver health [57, 79, 103].

### Lung cancer

The lung, historically considered a sterile environment, is now recognized as a complex microbial ecosystem. Emerging evidence suggests that dysregulation of microbiota plays a critical role in lung cancer pathogenesis, particularly through modulation of inflammatory responses [104–108]. This section examines the impact of lung microbiota dysbiosis on inflammation and explores the role of the oral microbiome in lung cancer progression. In patients with lung cancer, enrichment of lower airways with oral taxa, notably *Streptococcus* and *Veillonella*, associates with up-regulation of PI3K and ERK signaling in airway transcriptomes, linking community shifts to oncogenic pathway activation [109].

A homeostatic balance between the lung microbiota and the immune system is essential for maintaining pulmonary health [104]. Dysbiosis have been implicated in driving chronic inflammation, a well-established precursor to lung cancer [104–108]. In healthy lungs, commensal bacteria support immune tolerance and suppress excessive inflammatory responses [104]. However, an imbalance in the lung microbiome, characterized by a loss of beneficial bacteria and an overgrowth of pathogenic species, can shift the immune landscape toward a pro-inflammatory state that favors tumor development [104].

Several pathways have been proposed to explain the connection between lung dysbiosis and oncogenesis. Certain bacterial populations can stimulate inflammatory cytokine production, activating oncogenic pathways that promote the proliferation and survival of airway epithelial cells [107]. Studies have identified an enrichment of *Granulicatella*, *Abiotrophia*, and *Streptococcus* in lung cancer patients, coupled with reduced microbial diversity [107]. Mechanistically, oral-taxa enrichment in the lung is linked to Th17-skewed inflammation, with increased IL-17 signatures in bronchoalveolar samples from affected individuals [110]. In contrast, the depletion of *Staphylococcus* suggests a potential protective function for some bacterial species. Additionally, microbial dysbiosis has been linked to the activation of γδ T cells via an IL-1β/IL-23-dependent mechanism, leading to IL-17 secretion, neutrophil infiltration, and sustained inflammation within the tumor microenvironment [106, 107]. Furthermore, bacterial metabolites, such as N-formyl peptides, can recruit monocytes and neutrophils, exacerbating the inflammatory responses [106]. Pro-inflammatory cytokines, including IL-6 and IL-8, are frequently upregulated in dysbiotic states and have been shown to activate NF-κB signaling, a pathway known to enhance tumor cell proliferation, migration, and invasion [111, 112]. In contrast, the microbiota-derived metabolite desaminotyrosine (DAT) confers protection against influenza infection by amplifying type I interferon signaling, thereby mitigating immunopathology in the lungs [113].

The close anatomical relationship between the oral cavity and the lungs suggests that the oral microbiome could serve as a reservoir for bacterial species that influence lung cancer development [104, 105]. Alterations in the oral microbiota have been linked to systemic inflammation in patients with non-small cell lung cancer (NSCLC) and may serve as potential early indicators of disease, even among non-smokers [114, 115].

Specific oral bacterial taxa have been associated with lung tumorigenesis. For example, increased salivary levels of *Capnocytophaga* and *Veillonella* have been observed in lung cancer patients, suggesting their potential as diagnostic biomarkers [112]. Additionally, *Streptococcus* and *Veillonella* present in lung tissues have been linked to the activation of key oncogenic pathways, such as PI3K and ERK signaling, which contribute to cancer progression [106, 109]. One proposed mechanism through which the oral microbiome affects lung cancer is aspiration, whereby oral bacteria enter the lower respiratory tract [116]. Enrichment of supraglottic taxa in the lung microbiome has been correlated with heightened pulmonary inflammation, reinforcing a tumor-promoting environment [116].

### Ovarian cancer

The vaginal microbiota, primarily dominated by *Lactobacillus* species, plays a key role in maintaining vaginal health and modulating immune responses [117, 118]. Dysbiosis, characterized by a decline in *Lactobacillus* and increased microbial diversity, has been associated with ovarian cancer and risk factors such as BRCA1 mutations [117, 119]. High-throughput sequencing studies have revealed reduced microbial diversity in ovarian cancer tissues compared to normal distal fallopian tubes, with an increased presence of *Firmicutes* and *Proteobacteria* [117]. Additionally, a lower proportion of *Lactobacillus* in cervicovaginal samples has been linked to ovarian cancer [117, 120].

Dysbiosis may contribute to ovarian tumorigenesis by altering immune regulation [117]. Certain bacteria, such as *Gardnerella vaginalis* and *Lactobacillus iners*, can upregulate miRNA-223, potentially increasing ovarian cancer risk [121]. Differences among dominant Lactobacillus species are functionally important: *L. crispatus*–rich states produce more D-lactic acid and are linked to lower EMMPRIN/MMP-8 (matrix-remodeling) activity, whereas *L. iners*–dominant states are metabolically less acidic and more often accompany dysbiosis, which are features that can weaken barrier integrity and favor invasion-prone, pro-inflammatory milieus [122, 123]. In contrast, the depletion of vaginal microbiota in mouse models has been associated with reduced proliferation of Ki67-positive ovarian cancer cells [124]. A positive correlation between Toll-like receptor 2 (TLR2) levels in the cervicovaginal microenvironment and *Lactobacillus* dominance suggests a potential role of vaginal microbiota in antitumor immunity [119]. Consistent with this, immune-checkpoint protein profiles in the cervicovaginal microenvironment vary with microbiota: PD-L1 and LAG-3 inversely, and TLR2 positively, correlate with health-associated Lactobacillus dominance, linking local microbial states to immune-regulatory tone relevant to ovarian cancer immunosurveillance [125]. Furthermore, vaginal microbiota may influence cancer immunotherapy effectiveness, similar to the gut microbiome’s role in modulating responses to treatment [119].

Microbial metabolites produced by the vaginal microbiota can impact local and systemic metabolic pathways, potentially influencing hormone-driven cancers such as ovarian cancer [126]. Dysbiosis-associated metabolites, including those involved in lipid, amino acid, and short-chain fatty acid metabolism, may contribute to tumorigenesis [124]. For instance, enrichment of *Acinetobacter* and *Burkholderia* in vaginal dysbiosis has been linked to elevated glycerophospholipid and tryptophan metabolism, promoting ovarian cancer in murine models [124].

However, certain microbial species are beneficial. For example, *Lactobacillus*-deficient vaginal microbiota is more common in BRCA1/2 mutation carriers and are associated with an increased risk of ovarian cancer, suggesting that oncobiosis may exacerbate the impact of genetic predispositions [126]. Key *Lactobacillus* species, such as *L. lactis* and *L. reuteri*, help protect against ovarian cancer by suppressing pathogenic bacteria and modulating immune responses. *L. lactis*, for example, reduces CAOV-4 cell responsiveness to TLR4 activation by downregulating miR-21 and miR-200b expression [127–129] .

The gut microbiome also plays a role in estrogen metabolism by modulating β-glucuronidase activity, affecting estrogen circulation and regulation—key factors in ovarian cancer development [118]. While direct evidence linking vaginal microbial metabolites to ovarian cancer remains limited, established microbiome-hormone interactions in other malignancies suggest a possible role [126].

Despite increasing evidence associating vaginal microbiota to ovarian cancer, critical questions remain. The causal relationship between dysbiosis and ovarian tumorigenesis is unclear, requiring longitudinal studies to determine whether microbial alterations drive cancer development or result from tumor progression. While microbial metabolites and immune interactions are emerging as key factors, their precise roles require further elucidation. The potential for microbiota-based diagnostics and therapies, including probiotics or microbiome-targeted interventions, remains largely unexplored. Future research should prioritize validating microbial biomarkers for early detection and investigating microbiome-based strategies to enhance immunotherapy efficacy. Addressing these gaps will be essential for establishing the clinical relevance of vaginal microbiota in ovarian cancer, ultimately guiding novel diagnostic and therapeutic approaches.

### Skin cancer

As the largest organ of the human body and a primary barrier to the external environment, the skin functions as a complex ecosystem inhabited by diverse bacteria, viruses, and fungi. In addition to its general role in maintaining skin homeostasis and modulating disease outcomes, the cutaneous microbiota has been found to play a specific role in skin-related tumorigenesis [130]. Recent research has highlighted the intricate interactions between skin-resident microbes and the host immune system, revealing how these microorganisms can either suppress or promote cancer development.

Malignant melanoma (MM) is the most lethal form of skin cancer, accounting for approximately 75% of all skin cancer-related deaths. Emerging studies have provided evidence for the dual role of the skin microbiota in both promoting and inhibiting tumorigenesis [131]. In a 2020 study involving 27 patients with acral melanoma, researchers reported a significant association between the bacterial genus *Corynebacterium* and MM progression. Specifically, *Corynebacterium*-positive patients exhibited a higher proportion of IL-17-expressing immune cells compared to those without *Corynebacterium* colonization [132]. IL-17 has been shown to contribute to tumor growth by inducing the production of IL-6, which activates the oncogenic transcription factor STAT3 [133]. In a mouse model, *Corynebacterium* species consistently stimulated IL-17A production by Vγ4 + γδ T cells in the skin [134]. Collectively, these findings suggest that *Corynebacterium* may accelerate the development of malignant melanoma through the activation of an IL-17-dependent pathway. Conversely, a study in 2011 demonstrated that intratumoral administration of *Propionibacterium acnes* induces both local and systemic expression of Th1-type cytokines, including TNF-α and IFN-γ, thereby protecting the host from melanoma progression in a mouse model [135]. In addition to promoting tumor-suppressive cytokine responses, *P. acnes* has also been shown to reduce the risk of skin cancer by enhancing apoptosis in UVB-irradiated melanocytes [136]. Another commensal species, *Staphylococcus epidermidis*, has similarly exhibited anti-melanoma properties by stimulating the host immune response. *S. epidermidis* produce 6-HAP, a protein with selective antiproliferative activity against transformed tumor cells, contributing to the suppression of tumor growth [137]. 6-HAP is a selective DNA-synthesis inhibitor for transformed cells. Colonization with a 6-HAP-producing strain or systemic 6-HAP reduces UV-induced skin tumors and B16 melanoma growth in mice, linking a commensal-derived small molecule to direct tumor control [137]. Beyond MM, evidence also supports a close association between the microbiota and the development of other types of skin cancers. Notably, viral pathogens such as human herpesvirus-8 and human polyomavirus 5 have been causally linked to Kaposi sarcoma and Merkel cell carcinoma, respectively [138, 139].

Taken together, these findings underscore the dynamic and context-dependent role of the microbiota in skin cancer. Depending on its composition and interaction with the host immune system, the cutaneous microbiota can act either as a protective agent or as a potential promoter of tumorigenesis. Continued investigation into these microbial-host interactions may lead to the development of novel microbiota-based strategies for the diagnosis, prevention, and treatment of skin cancers.

### Gastric cancer

*Helicobacter pylori* is a Gram-negative bacterial pathogen that selectively colonizes the gastric epithelium and has coevolved with humans for tens of thousands of years. Globally, more than half of the population is colonized by *H. pylori*, and in most individuals, this leads to chronic gastritis. Persistent infection has been identified as a significant risk factor for gastric adenocarcinoma, with epidemiological studies—such as one involving 1,526 Japanese patients—demonstrating that gastric cancer developed in approximately 3% of *H. pylori*-infected individuals, compared to none among the uninfected cohort [140].

The oncogenic potential of *H. pylori* arises from a multifactorial interplay between bacterial virulence determinants, host immune response, epigenetic reprogramming, and environmental modifiers. Central among these virulence factors is the cytotoxin-associated gene A (CagA), which is delivered into host gastric epithelial cells via a type IV secretion system encoded by the cag pathogenicity island (cag PAI) [141–143]. Once phosphorylated, CagA aberrantly activates host signaling pathways—including those involving SHP-2, ERK1/2, and β-catenin—inducing cellular elongation, increased motility, and proliferation, all of which are hallmarks of early neoplastic transformation [144]. Even unphosphorylated CagA disrupts epithelial junctions, perturbs polarity, and activates inflammatory and mitogenic cascades [145–151]. In vitro and in vivo studies further reveal that CagA mediates epigenetic silencing of tumor suppressive microRNAs, including let-7, miR-26a, and miR-101, through CagA-induced upregulation of c-Myc, DNMT3B, and EZH2, leading to Ras pathway activation [152]. These changes are observed in CagA transgenic mice and occur even in the absence of inflammation, underscoring the direct oncogenic role of CagA.

In addition, genome-wide methylation analyses have identified FOXD3 as a key epigenetic target during *H. pylori*-associated gastric tumor progression. Hypermethylation of the FOXD3 promoter occurs both in mice and human tumors and correlates with decreased survival in patients with gastric cancer. FOXD3 silencing reduces the expression of CYFIP2 and RARB, pro-apoptotic genes that FOXD3 directly activates. Functional studies show that restoring FOXD3 expression suppresses gastric cancer cell proliferation, invasion, and tumor growth in xenograft models, in part by promoting apoptosis. Together, these findings reveal that *H. pylori*—through CagA and downstream epigenetic regulators—reprograms host gene expression to drive carcinogenesis [153].

Other virulence factors further contribute to gastric pathology. The cag PAI can translocate peptidoglycan fragments, activating NOD1 and NF-κB-mediated proinflammatory signaling. This NOD1 sensing requires cag T4SS-mediated peptidoglycan delivery into epithelial cells and is a well-defined trigger of NF-κB/IL-8 responses during carcinogenic gastritis [154]. VacA, another key toxin, alters epithelial integrity, immune signaling, and induces apoptosis. VacA also exerts immunosuppressive effects by inhibiting T-cell proliferation/IL-2 production and by disrupting mitochondrial dynamics to induce apoptosis, mechanisms that can blunt anti-tumor immunity in the gastric mucosa [155, 156]. Moreover, *H. pylori*’s adhesins and outer membrane proteins (OMPs)—including BabA and OipA—facilitate colonization, persistence, and inflammation [157, 158].

In contrast, certain bacteria exert anti-gastric cancer effects through several mechanisms, including releasing bioactive substances, depleting nutrients essential for tumor metabolism and proliferation, enhancing host immunity, and beyond [159–161]. For instance, LPS in the outer membrane of Gram-negative bacteria activates the host's cell-surface TLR4, thereby triggering a T cell-mediated response against tumor cells [159, 162]. *L. casei* produces ferricrome, which triggers apoptosis in tumor cells by activating the c-Jun N-terminal kinase (JNK) pathway and subsequently induces DNA damage-inducible transcript 3 (DDIT3) exprssion in cancer cells [159, 163]. Host genetic and immunological factors further modulate disease risk. Polymorphisms in IL1B, TNFA, and IL8 promote inflammation and acid suppression, creating a carcinogenic niche. Conversely, IL10 polymorphisms linked to reduced anti-inflammatory response also contribute to risk. A persistent Th1/Th17-skewed immune response and diminished Treg control drive chronic inflammation, while metabolic reprogramming in macrophages, including polyamine-mediated NO suppression and apoptosis, further weakens mucosal defense and facilitates tumor development [151].

## Microbiome-immune interactions in cancer immunity

**Fig. 2:**
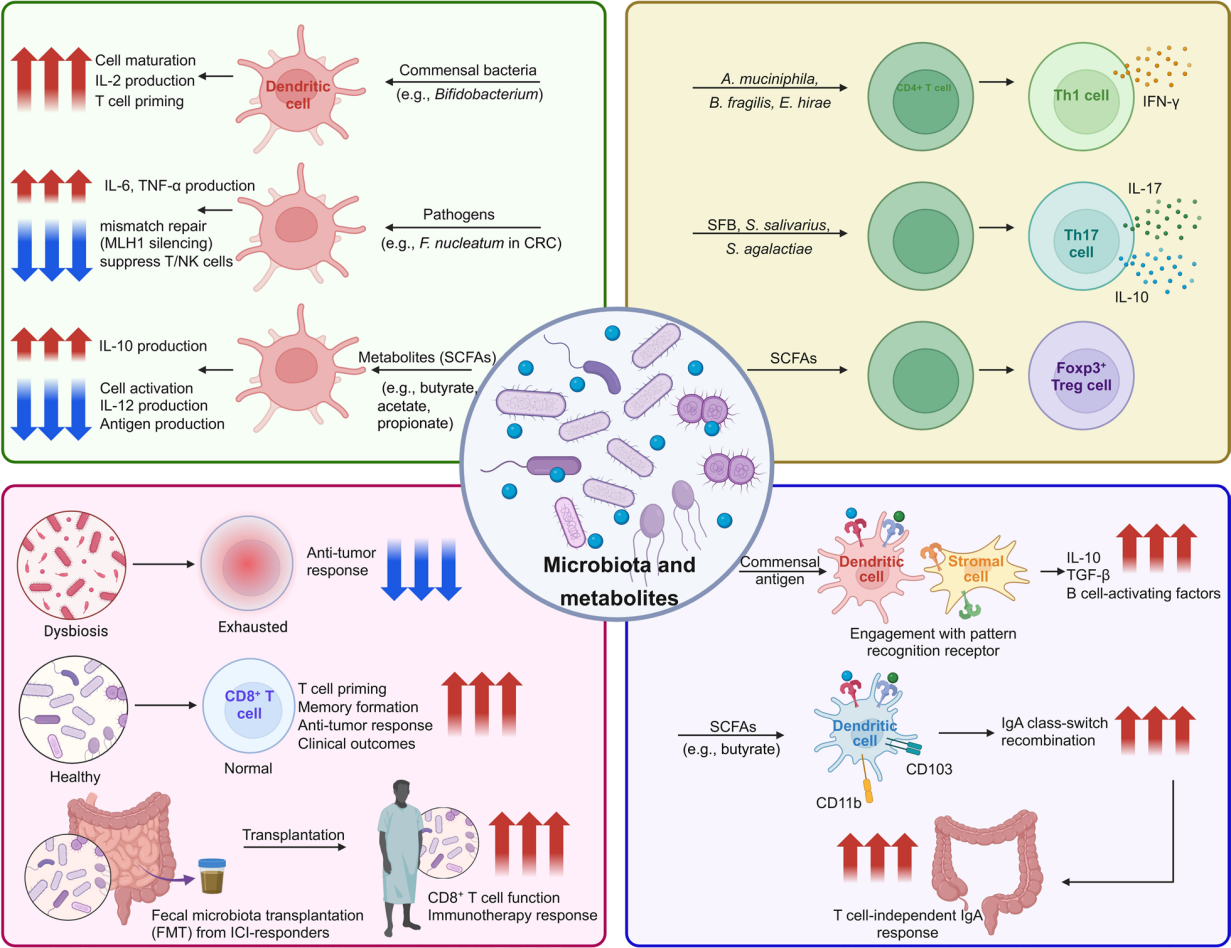
Microbiota-immune interactions in cancer immunity. This diagram depicts the microbiota–immune interactions in the context of cancer immunity from four perspectives: dendritic cells, CD4⁺ T cells, CD8⁺ T cells, and antibody responses. Microbial products and metabolites shape dendritic cell maturation, cytokine production, and antigen presentation. These cues direct CD4⁺ T cell differentiation into effector and regulatory subsets and influence CD8⁺ T cell priming, effector function, and memory formation. In the humoral compartment, commensals promote B cell development, class-switch recombination, and antibody production, especially IgA at mucosal sites. While some microbes enhance antitumor IgG responses, others drive regulatory IgA⁺ B cells that suppress cytotoxic T cells and foster immune evasion. Together, these multifaceted interactions shape cancer immunity and therapeutic outcomes

Microbiome contribute to the maintenance of immune homeostasis by regulating both innate and adaptive immune responses (Fig. 2). Commensal bacteria interact with the host's immune system to promote the development and function of immune cells [164]. Conversely, microbial imbalances, or dysbiosis, can disrupt this equilibrium, leading to chronic inflammation and creating an environment conducive to tumor development [165]. Understanding the intricate interplay between the microbiome and the immune system is therefore critical for developing novel therapeutic strategies aimed at harnessing the microbiome to improve cancer prevention and treatment outcomes.​

### Dendritic cells (DCs) responses and antigen presentation

DCs are pivotal antigen-presenting cells that bridge innate sensing to adaptive immunity, and emerging evidence indicates that the microbiome profoundly shapes DC function in the tumor microenvironment​ [166, 167]. Microbial signals, from gut commensals as well as bacteria within tumors, can influence DC maturation status, cytokine production, and antigen-presenting capacity. This modulation can tilt DCs toward either immunogenic or tolerogenic phenotypes, affecting whether tumor antigens stimulate effective cytotoxic T lymphocytes or instead induce regulatory or anergic T [166, 168–170]. In cancers across different organ sites, microbiome-derived molecules have been shown to alter the frequency of various DC subsets in tumors and draining lymph nodes, the expression of key co-stimulatory or MHC molecules on DCs, and the cytokines that mediate T cell differentiation [171]​.

SCFAs such as butyrate, propionate, and acetate are prominent microbial metabolites with immunomodulatory effects. SCFAs generally tend to dampen DC activation by interfacing with metabolic and epigenetic pathways in these cells. For example, butyrate can suppress the glycolytic burst and mTOR signaling needed for full DC maturation. Functionally, DCs exposed to SCFAs during their activation acquire a more tolerogenic profile, characterized by reduced production of pro-inflammatory cytokines like IL-12 and enhanced secretion of IL-10​ [172, 173]. Excess butyrate has been shown to impair DC-mediated tumor antigen cross-presentation: in a melanoma model, antibiotic depletion of butyrate-producing gut commensals led to heightened DC cross-priming of tumor antigens and stronger CD8^+^ T cell responses after radiotherapy, whereas reintroducing butyrate abrogated this effect [172]​.

Commensal microbes can profoundly affect how efficiently tumor antigens are presented by DCs to T cells. For instance, a study demonstrated that mice harboring *Bifidobacterium* had superior DC activity in tumors, leading to enhanced CD8^+^ T cell priming and accumulation, with tumor control comparable to checkpoint blockade therapy​ [174]. In this study, oral administration of *Bifidobacterium* alone improved DC-dependent T cell responses against melanoma, and combining this probiotic with PD-L1 blockade nearly abolished tumor growth​. The microbiome-driven boost in DC function, reflected by increased maturation and IL-12 production, was identified as the mediator of this effect​ [174]. Moreover, in another experiment, fecal transplant from anti-PD-1 therapy responders into mice improved anti-tumor efficacy in an IL-12-dependent manner, highlighting the critical role of microbiome-induced DC IL-12 in the therapeutic immune response​ [171].

In contrast, a dysbiotic or pathogenic microbiome may impair DC antigen presentation and promote immune evasion. For example, in CRC, pathogens like *F. nucleatum* can abundantly colonize tumors and interact with myeloid cells. *Fusobacterium* infection triggers DCs or macrophages to release IL-6, TNFα and other inflammatory mediators, thereby fostering a pro-inflammatory environment that epigenetically silences MLH1, reduces the enzymatic activity of mismatch repair proteins, and inhibits T and natural killer (NK) cells [5, 166, 175]. In addition, *F. nucleatum* virulence factors inhibit T cell proliferation [176, 177]. In such case, DCs influenced by the microbiome skew toward driving pro-tumorigenic immune subsets rather than protective responses.

### CD4⁺ T cell responses

The gut plays a pivotal role in shaping the differentiation of CD4⁺ T cell subsets, including Th1, Th2, and Th17 cells [178–180]. Under optimal conditions, the microbiome contributes to immune regulation and pathogen clearance. However, dysbiosis, an imbalance in the microbial community, has been linked to various inflammatory disorders, including cancer [181]. This disruption alters the signals driving CD4⁺ T cell differentiation, potentially skewing immune responses towards either pro- or anti-tumorigenic outcomes.

Animal model studies have provided valuable insights into the microbial influences on Th cell differentiation. For instance, segmented filamentous bacteria (SFB) are well-documented inducers of Th17 responses in the gut, which can extend to distal sites such as the central nervous system, influencing autoimmune disease progression [179, 182]. In the context of non-small cell lung cancer, patients exhibited increased frequencies of Th1 and Th17 cells reactive to Streptococcus salivarius and S. agalactiae in peripheral blood, lung cancer tissue, and the gastrointestinal tract compared to healthy controls [181]. Notably, responses to *E. coli*, a common gut bacterium, were not upregulated in lung cancer tissue, suggesting antigen-specific immune activation. This response appears to be mediated by MHC class II interactions, with *S. salivarius* and *S. agalactiae* also stimulating monocytes to secrete Th1- and Th17-skewing cytokines such as IL-6, IL-12, and TNF [181].

Moreover, microbiome diversity itself plays a role in shaping CD4⁺ T cell responses, prompting research into how complex microbial communities regulate mucosal immunity [179]. Metabolic products of the microbes, such as short-chain fatty acids (SCFAs), further influence CD4⁺ T cell differentiation, often promoting regulatory T cells (Tregs) and immune homeostasis [180, 183]. Microbiota-reactive CD4⁺ T cells are key sources of cytokines within the TME and systemically, impacting tumor progression. IL-17, produced by Th17 cells, accumulates in many tumors with dual roles in cancer immunity [184, 185].

While IL-17 promotes tumor growth through angiogenesis and immunosuppressive functions, it can also enhance anti-tumor immunity by recruiting immune cells and activating CD8⁺ T cells [184]. The TME itself modulates Th17 plasticity, with chemokines such as CCL20, CCL17, and CCL22 recruiting Th17 cells, while tumor-derived cytokines such as IL-1β, IL-6, IL-23, and TGF-β promote their differentiation [186, 187]. In NSCLC, heightened Th1 and Th17 responses to specific bacteria were associated with monocyte-driven secretion of pro-inflammatory cytokines, reinforcing the role of the microbiome in fostering a pro-inflammatory TME [181]. IFN-γ, a key Th1 cytokine, is essential for anti-tumor immunity and is influenced by the gut. IFN-γ-producing CD4⁺ T cells reactive to *Akkermansia muciniphila*, *Bacteroides fragilis*, and *Enterococcus hirae* correlate with favorable clinical outcomes in cancer patients [188]. The microbiome also modulates the Th17/Treg balance within the TME, influencing cytokine production that can either promote inflammation and anti-tumor immunity (e.g., IFN-γ, IL-17) or suppress immune responses (e.g., IL-10 from Tregs or regulatory Th17 cells) [179, 182]. A microbiome favoring Treg expansion may facilitate tumor progression by dampening cytotoxic and Th1 responses [180, 183, 188].

Beyond effector T cell differentiation, the microbiome significantly impacts the development and expansion of Foxp3⁺ Tregs [179, 180]. Tregs maintain immune homeostasis but can also suppress anti-tumor immunity, depending on the context. SCFAs, generated through bacterial fermentation of dietary fiber, promote peripheral Treg differentiation in the colon, suggesting that dietary interventions could influence Treg accumulation in the TME [180, 183, 188]. However, microbiome-driven immune responses are not strictly pro- or anti-inflammatory; Th17 cells induced by commensal bacteria can acquire regulatory functions, producing IL-10 to suppress effector T cell activity. In intestinal homeostasis, commensal-specific Th17 cells exhibit an anti-inflammatory phenotype, dependent on c-MAF and IL-10, further demonstrating the plasticity of microbiome-specific immune responses [182]. The balance between Treg and effector T cell populations, regulated by the microbiome, is crucial in determining anti-tumor immunity outcomes.

The balance between microbiome-driven pro- and anti-inflammatory responses is evident across different cancers. In NSCLC, specific bacterial phyla have been linked to improved survival in patients treated with immune checkpoint inhibitors [181]. Microbiome-targeting interventions, such as FMT or antibiotics, offer potential strategies for modulating anti-tumor immunity. For example, FMT from long-term pancreatic cancer survivors into mice enhanced CD8⁺ T cell activation and tumor control [188, 189]. Conversely, antibiotic treatment has demonstrated both positive and negative effects, depending on its impact on immunosuppressive bacterial populations.

### CD8⁺ T cell responses

The gut microbiota plays a pivotal role in shaping CD8⁺ T cell responses, influencing their activation, memory formation, and anti-tumor function [188]. A diverse and balanced microbiota is essential for robust CD8⁺ T cell immunity, as demonstrated by studies in germ-free (GF) mice, where the absence of microbiota results in defective memory T cell formation and weakened recall responses upon antigen re-exposure [188, 190]. This suggests that continuous microbial signaling supports optimal CD8⁺ T cell memory and persistence. Conversely, microbial dysbiosis has been associated with both chronic activation and exhaustion of CD8⁺ T cells, impairing their tumor-killing capacity [188, 191]. For instance, in a mouse model of inflammation-associated tumorigenesis, dysbiosis initially drove an increase in colonic CD8⁺ IFNγ⁺ T cells, but these cells later became functionally exhausted within the tumor microenvironment (TME), reducing their efficacy against tumors [191]. Moreover, the composition of the gut microbiota has been strongly associated with cancer immunotherapy outcomes, particularly immune checkpoint inhibitors (ICIs) [191–193]. Fecal microbiota transplantation (FMT) studies have shown that transferring microbiota from ICI-responsive patients to germ-free or antibiotic-treated mice enhances tumor control, likely by modulating CD8⁺ T cell responses [194]. These findings underscore the gut microbiota’s role as a critical regulator of systemic anti-tumor immunity.

Beyond influencing overall immune landscapes, specific microbial metabolites directly modulate CD8⁺ T cell effector functions [188, 194]. Short-chain fatty acids (SCFAs)—including butyrate, acetate, and propionate—play a central role in shaping T cell metabolism and function [190]. SCFAs also enhance CD8⁺ T cell cytotoxicity by increasing IFN-γ production and promoting the expression of effector molecules like TNF-α [188, 194]. Butyrate, for example, promotes oxidative phosphorylation and enhances the memory potential of activated CD8⁺ T cells by modulating metabolic pathways, such as uncoupling the tricarboxylic acid cycle from glycolytic input [190]. It has been shown to inhibit DNA binding protein 2 (ID2)-dependent IL-12 signaling, further enhancing CD8⁺ T cell-mediated tumor clearance [194]. Additionally, microbial-derived metabolites such as inosine provide an alternative carbon source in glucose-restricted tumor microenvironments, sustaining T cell function and improving ICI efficacy [194]. In contrast, certain microbial byproducts, such as deoxycholic acid (DCA), a secondary bile acid, can suppress CD8⁺ T cell effector function by interfering with calcium signaling [195]. These findings highlight how microbial-derived signals fine-tune CD8⁺ T cell metabolism and function, directly shaping their capacity to eliminate tumor cells.

### Antibody responses

The commensal microbiota profoundly influences host humoral immunity, extending its impact to cancer contexts. Under homeostatic conditions, microbiota-driven signals are essential for normal B cell development and antibody production, especially at mucosal sites. Germ-free or antibiotic-treated mice have underdeveloped Peyer’s patches, reduced immunoglobulin A (IgA) secretion, and general B cell defects​, underscoring that microbial exposure is crucial to the antibody system [196]. Mechanistically, commensal antigens engage pattern recognition receptors on dendritic cells and stromal cells, triggering the release of B cell-activating factors and cytokines that drive Ig class-switch recombination (CSR) [197]. Gut and lung microbiota can promote IgA CSR through CD103^+^ DC antigen presentation and the production of IL-10 and TGF-β [197, 198]​. In turn, commensal-derived metabolites like SCFAs amplify this process. For example, butyrate increased TGF-β production by CD103^+^CD11b^+^ DCs, thereby promoting a T-cell-independent IgA response in the colon [199]. Notably, dysbiosis can skew this IgA-microbiota axis and thereby affect cancer risk and immunity​ [54, 192, 200]. For instance, butyrate can stimulate regulatory B cells to increase IL-10 expression via the aryl hydrocarbon receptor, driving somatic hypermutation and unbalanced IgA production that may facilitate tumor growth [201–203]. These findings reveal that modulating the microbiota can reshape the host's humoral immune repertoire, thereby influencing antitumor immunity through changes in antibody production.

Recent studies have demonstrated that commensal microbes can modulate antitumor antibody responses. In CRC, introducing a single immunogenic bacterium can boost local humoral immunity: colonization of mice with *Helicobacter hepaticus* stimulated specific T follicular helper cells, expanded tumor-infiltrating B cells, and promoted the maturation of tertiary lymphoid structures adjacent to tumors​ [204]. Moreover, an early-phase trial in solid tumors showed that oral administration of Microbial Ecosystem Therapeutic 4 (MET4), a consortium of 30 cultivated gut bacterial species derived from healthy donor stool, could induce circulating anti-MET4 IgG antibodies [205]. High-IgG responders in this trial exhibited an expansion of multiple gut taxa previously linked to immunotherapy success, such as *Collinsella aerofaciens*, and showed increased circulating B cell frequencies ​[205].

On the other hand, tumors can employ microbiota-driven B cell responses to favor immune escape. As mentioned, mucosal microbes induce IgA production. Elevated binding of IgA without defined function blocks Fcα receptors on macrophages, neutrophils, and NK cells​, thereby impeding antibody-dependent cellular cytotoxicity or phagocytosis [206, 207]. Multiple studies have identified IgA^+^ tumor-infiltrating B cells as a regulatory subset that dampens anti-tumor immunity as these IgA^+^ cells often express molecules directly exhaust CD8^+^ T cells and expand regulatory T cells​. In mouse models of pancreatic cancer, the majority of intratumoral B cells induced by chemotherapy were IgA^+^ and highly immunosuppressive, and depleting these IgA^+^ cells was required for successful tumor eradication [208]. In hepatocellular and prostate tumors, IgA^+^ cells suppress CD8^+^ responses and induce immunosuppressive TME through high levels of PD-L1, IL-10, and TGF-β expression [209–211]. Clinically, patients with IgA-producing B cell infiltration tend to have poorer survival in CRC and melanoma, and high level of intratumoral IgA associates with shorter survival in bladder cancer​ [197, 212–215]. The dual role of antibodies in cancer highlights the need to better understand the microbial cues that drive responses toward either protective or immunosuppressive outcomes.

### Granulocytes as microbiome-educated effectors

Commensal cues calibrate neutrophil tone at baseline: circulating peptidoglycan sensed by NOD1 systemically primes bone marrow-derived neutrophils for enhanced killing, while the microbiota also governs neutrophil aging via TLR-MyD88 pathways, features that influence inflammatory readiness and the propensity for NETosis under stress [216–219]. Dysbiosis and inflammatory stimuli can then amplify neutrophil extracellular trap (NET) formation. NETs physically capture circulating tumor cells and promote metastatic outgrowth, effects that are reversible with DNase or neutrophil elastase inhibition [220, 221]. In CRC, NETs correlate with invasion and liver metastasis and can drive metastatic signaling in preclinical models [222, 223]. Notably, *F. nucleatum* directly triggers NETosis in neutrophils, providing a microbe-level mechanism that can accelerate tumor progression [224, 225].

Eosinophils exhibit context-dependent roles that are tunable by the microbiome and epithelial alarmins such as IL-33. At baseline, germ-free mice display exaggerated small-intestinal eosinophilia that normalizes upon colonization, indicating that resident microbes restrain eosinophil accumulation and behavior [226, 227]. Microbial SCFAs further act as metabolic levers. Butyrate directly dampens eosinophil chemotaxis, and SCFA supplementation or high-fiber diets reduce eosinophilic inflammation in vivo, supporting a diet-microbiota route to tune eosinophil effector thresholds [228–230]. The IL-33 axis is also microbiota-responsive: dysbiosis can elevate epithelial IL-33, a potent chemoattractant/activator for eosinophils and type 2 innate lymphoid cells, linking microbial perturbations to eosinophil recruitment and activation at mucosal sites [231, 232]. In tumors, eosinophils can normalize aberrant vasculature and promote CD8⁺ T-cell infiltration, changes that associate with rejection, so microbiome-driven shifts in SCFAs and IL-33 provide plausible mechanisms by which commensals bias eosinophils toward anti-tumor functions in selected settings [233].

Basophils, although less studied in oncology, do infiltrate human tumors and can shape Th2-type environment with context-dependent clinical associations. In pancreatic ductal adenocarcinoma, basophil recruitment to tumor-draining lymph nodes is associated with Th2 inflammation and independently predicts poorer postsurgical survival [234, 235]. By contrast, in other cohorts, including NSCLC and soft-tissue sarcoma, higher basophil counts or activated basophil gene signatures have correlated with better outcomes or treatment response, underscoring disease- and context-specific effects [236–238]. Importantly, early-life and compositional features of the microbiota calibrate systemic IgE set-points that, via high-affinity FcεRI on basophils, tune their development and activation potential, providing a mechanistic bridge between microbes and basophil-driven tumor immunity [239, 240].

## Role of microbiome in cancer immunotherapy

**Fig. 3:**
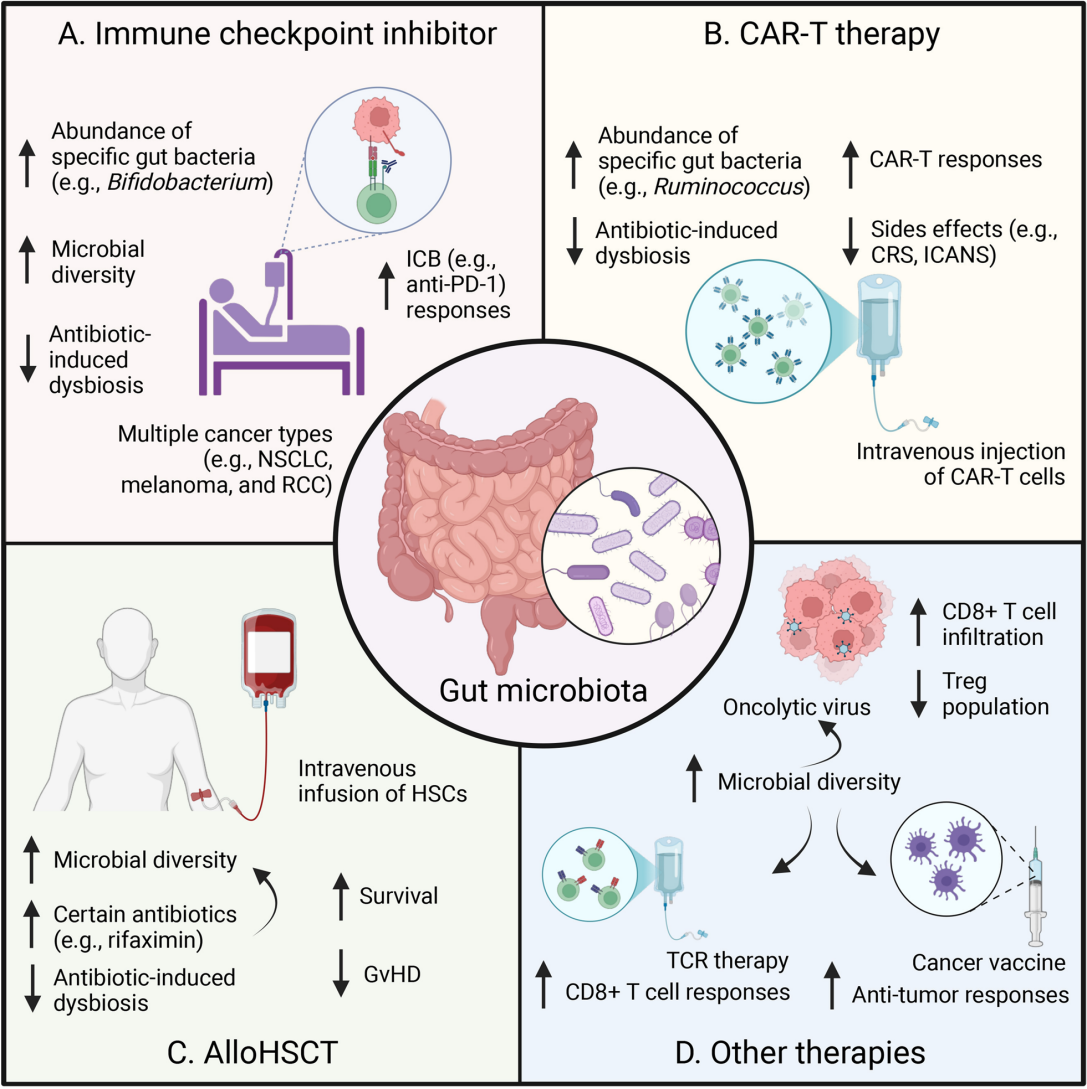
Microbiota influence on cancer immunotherapy. **A** ICB Therapy: Beneficial gut bacteria enhance ICB responses while dysbiosis from antibiotics reduces efficacy. **B** CAR-T Therapy: Specific microbes improve CAR-T responses and reduce side effects such as CRS and ICANS. Dysbiosis impairs CAR-T cell expansion and function. **C** AlloHSCT: **A** diverse microbiome supports better survival and reduced GVHD in AlloHSCT. Antibiotic-induced dysbiosis worsens transplant outcomes. **D** Other Therapies: Microbial diversity enhances responses to cancer vaccines, TCR therapies, and oncolytic viruses by promoting T-cell activation and antitumor immunity. *ICB* immune checkpoint blockade, *PD-1* programmed cell death protein 1, *CAR-T* Chimeric Antigen Receptor T-cell, *CRS* cytokine release syndrome, *ICANS* immune effector cell-associated neurotoxicity syndrome, *AlloHSCT* allogeneic hematopoietic stem cell transplantation, *GVHD* graft-versus-host disease, *TCR* T-cell receptor

Emerging evidence suggests that specific microbial species and their metabolites can significantly influence the efficacy of cancer immunotherapies (Table 1), such as ICB, by modulating the tumor microenvironment and systemic immune responses (Fig. 3) [241]. Similarly, while specific bacterial species have been associated with enhanced antitumor immunity, dysbiosis-induced disruptions of the microbiome have been linked to diminished therapeutic efficacy [194].

**Table 1:** Clinical trials modulating microbiota in cancer immunotherapy

Disease indication	Microbiota modulation	Immunotherapy	Current stage	ClinicalTrials.gov identifier
PD-1 resistant/refractory melanoma	FMT from immunotherapy responders through a colonoscopy	Pembrolizumab	Completed	NCT03341143
Metastatic melanoma	FMT from immunotherapy responders through a colonoscopy and oral administered stool capsules	Anti-PD-1	Unknown status	NCT03353402
Melanoma	FMT from healthy donor through oral administered capsules	Pembrolizumab/nivolumab	Active, not recruiting	NCT03772899
Melanoma or genitourinary cancer	FMT from healthy donors	ICIs	Recruiting	NCT03819296
Genitourinary cancer	FMT through a colonoscopy	Loperamide	Recruiting	NCT04038619
Metastatic mesothelioma	FMT from a healthy family donor through a colonoscopy	Anti-PD-1	Completed	NCT04056026
Metastatic castration resistant prostate cancer	FMT from enzalutamide-pembrolizumab responders through an endoscopy	Pembrolizumab	Unknown status	NCT04116775
Gastrointestinal cancer	FMT from healthy donors through capsules	Anti-PD-(L)1	Completed	NCT04130763
Renal cell carcinoma	FMT from healthy donors through capsules	Nivolumab and ipilimumab	Active, not recruiting	NCT04163289
Advanced solid tumor	FMT from immunotherapy responders through a colonoscopy	Immunotherapy	Unknown status	NCT04264975
Metastatic or inoperable melanoma, microsatellite instability-high or mismatch-repair deficient cancer, or non-small cell lung cancer	FMT from immunotherapy responders through fecal capsules	Nivolumab	Unknown status	NCT04521075
Melanoma	FMT from prior malignant melanoma patients in remission for at least 1 year through stool	ICIs	Terminated	NCT04577729
Metastatic colorectal cancer	FMT from healthy donors through capsules	Pembrolizumab or nivolumab	Active, not recruiting	NCT04729322
Renal cell carcinoma	FMT from immunotherapy responders through frozen fecal capsules	ICIs	Active, not recruiting	NCT04758507
Any malignant indication	FMT from a healthy family donor through a colonoscopy	ICIs	Active, not recruiting	NCT04883762
Advanced lung cancer	FMT from 3 donors selected based on the fecal abundance of bacterial taxa through capsules	Anti-PD-1	Completed	NCT04924374
Non-small cell lung cancer and melanoma	Investigational FMT through capsules	Pembrolizumab and ipilimumab/nivolumab	Active, not recruiting	NCT04951583
Unresectable or metastatic melanoma	FMT from pooled donors through an enema	Ipilimumab and nivolumab	Recruiting	NCT04988841
Non-small cell lung cancer	FMT through capsules containing washed fecal microbiota	Anti-PD-1/PDL-1	Unknown status	NCT05008861
Refractory metastatic melanoma	FMT from anti-PD-1 therapy responders through esophagogastroduodenoscopy	Anti-PD-1	Recruiting	NCT05251389
Refractory malignancy	FMT from immunotherapy responders through a colonoscopy	ICIs	Completed	NCT05273255
Metastatic or locally advanced colorectal adenocarcinoma	FMT through capsules	Sintilimab and fruquintinib	Unknown status	NCT05279677
Malignant melanoma, non-small cell lung cancer, cutaneous squamous cell carcinoma, head and neck squamous cell carcinoma, renal clear cell carcinoma or microsatellite instability-high solid cancer	FMT from immunotherapy responders	ICIs	Recruiting	NCT05286294
Metastatic lung cancer	FMT from immunotherapy complete responders through oral administered capsules	ICIs	Recruiting	NCT05502913
Advanced hepatocellular carcinoma	FMT through capsules	Atezolizumab and bevacizumab	Not yet recruiting	NCT05690048
Advanced hepatocellular carcinoma	FMT from PD-(L)1-based immunotherapy responders	Atezolizumab and bevacizumab	Recruiting	NCT05750030
Advanced melanoma	EPD1503: an orally available preparation derived from a single clone *of Bifidobacterium spp.*	Pembrolizumab	Active, not recruiting	NCT03595683
Non-small cell lung cancer, renal cell carcinoma, bladder cancer or melanoma	MRx0518: a live biotherapeutic product consisting of a lyophilised formulation of a proprietary strain of bacterium	Pembrolizumab	Terminated	NCT03637803
Solid tumors	MET-4: an oral alternative to fecal transplant consisting a mixture of intestinal bacteria isolated from healthy donor stool sample	Anti-PD-1/PD-L1	Active, not recruiting	NCT03686202
Advanced metastatic colorectal carcinoma, triple-negative breast cancer, and checkpoint inhibitor relapsed tumors	EPD1503: an orally available preparation derived from a single clone *of Bifidobacterium spp.*	Pembrolizumab	Completed	NCT03775850
Unresectable or metastatic melanoma	SER-401: a combination of yet not disclosed microbes	Nivolumab	Completed	NCT03817125
Stage IV or advanced kidney cancer	CBM588: a probiotic containing a specific strain of *Clostridium butyricum*	Nivolumab and ipilimumab	Completed	NCT03829111
Advanced or metastatic cancer	VE800: a combination of eleven nonpathogenic, nontoxigenic, human commensal bacterial strains isolated from healthy human donor stool samples	Nivolumab	Completed	NCT04208958
Advanced or metastatic solid tumors	GEN-001: a single *Lactococcus lactis* strain isolated from the gut of a healthy human volunteer	Avelumab	Completed	NCT04601402
Resectable non-small cell lung cancer	BiFico: a type of probiotic mixture containing *Bifidobacterium*, *Lactobacillus acidophilus*, and *Enterococcus faecalis*	Nivolumab	Active, not recruiting	NCT04699721
Non-small cell lung cancer	MS-20: a postbiotic composed of microbial metabolites derived from a soybean-based medium fermented with probiotics and yeast strains	Pembrolizumab	Completed	NCT04909034
Liver cancer	Probio-M9: a *Lactobacillus rhamnosus* probiotic strain	Anti-PD-1	Unknown status	NCT05032014
Non-small cell lung cancer	Kex02: *Lactobacillus Bifidobacterium* V9	Carilizumab	Unknown status	NCT05094167
Advanced or metastatic kidney cancer	CBM588: a probiotic containing a specific strain of *Clostridium butyricum*	Nivolumab and Cabozantinib	Active, not recruiting	NCT05122546
Urothelial bladder carcinoma	Live combined *Bifidobacterium*, *Lactobacillus* and *Enterococcus* capsules	Immunotherapy	Unknown status	NCT05220124
Metastatic or locally advanced unresectable clear cell renal cell carcinoma, cutaneous melanoma, or EGFR/ ALK wildtype adenocarcinoma-type non-small cell lung carcinoma	BMC128: a live bacterial consortium of 4 bacterial strains	Nivolumab	Active, not recruiting	NCT05354102
Liver cancer	*Bifidobacterium bifidum* oral product	Carrilizumab and apatinib mesylate	Unknown status	NCT05620004
Non-small cell lung cancer	Yeast derived β-glucan	Vaccine 1650-G	Completed	NCT01829373
Solid cancer	Bob's Red Mill potato starch	Ipilimumab and nivolumab	Completed	NCT04552418
Metastatic renal cell carcinoma	Camu Camu prebiotic	Ipilimumab and nivolumab	Recruiting	NCT06049576
Malignant neoplasm except small cell neuroendocrine tumors	Five-day plant-based, low-calorie, low-protein, and low carbohydrate fasting-mimicking diet	Standard-of-care treatment including immunotherapy	Completed	NCT03340935
Solid or hematologic tumors	Prolon by L-Nutra: a medically-designed fasting mimicking dietary kit	Trastuzumab/pertuzumab/cetuximab/bevacizumab/nivolumab/pembrolizumab	Completed	NCT03595540
Non-small cell lung cancer	Chemolieve: a plant-based fasting mimicking diet	Pembrolizumab	Completed	NCT03700437
LKB1-inactive lung adenocarcinoma	Five-day fasting-mimicking diet consisting of 700 KCal on day 1, 300 KCal on days 2–4, and 450 KCal on day 5, to be repeated every three weeks up to a maximum of 4 cycles	Pembrolizumab	Unknown status	NCT03709147
Metastatic renal cell carcinoma	Ketogenic diet 2:1	Nivolumab and ipilimumab, pembrolizumab and axitinib, sunitinib or pazopanib	Completed	NCT04316520
Stage III-IV melanoma	Dietary Intervention: isocaloric whole foods diet higher in fiber	Pembrolizumab or nivolumab	Active, not recruiting	NCT04645680
Melanoma	High fiber, plant based diet and exercise prescription with acceptance and commitment training sessions	Ipilimumab and nivolumab, relatlimab and nivolumab, pembrolizumab, or nivolumab	Recruiting	NCT04866810
Metastatic renal cell carcinoma	Low-carbohydrate and high-fat diets/ liquid ketone supplement β-hydroxybutyrate monoester	Nivolumab and ipilimumab	Terminated	NCT05119010
Advanced head and neck cancer	Prolonged Nightly Fasting: limiting daily period of food intake to 10 h and nightly fasting period to 14 h	Nivolumab, pembrolizumab, atezolizumab, avelumab or durvalumab	Completed	NCT05083416
Solid tumor malignancies	Control diet consisting of 20% protein or intervention diet consisting of 10% protein	ICIs	Recruiting	NCT05356182
Metastatic non-small cell lung cancer	Immunonutrition: additional two servings of an oral high-calorie-high-protein nutritional liquid supplement enriched in Oral Impact immunonutrients	Immunotherapy	Recruiting	NCT05384873
Extensive-stage small-cell lung cancer	Cyclic, 5-day calorie restriction: about 600 kcal on day 1, 300 kcal on days 2 to 5, plant-based, low-protein, low-carbohydrate diets	Atezolizumab	Not yet recruiting	NCT05703997
Triple negative breast cancer	Fasting-Like Approach: a plant-based, low-calorie (about 600 kcal on day 1, 300 kcal on day 2 to 5), low-protein, low-carbohydrate diet	Pembrolizumab	Recruiting	NCT05763992
Refractory primary hepatocellular carcinoma or liver dominant metastatic cancer from colorectal or pancreatic cancers	Oral vancomycin	Nivolumab and oral tadalafil	Completed	NCT03785210

### ICB therapy

Accumulating evidence indicates that the gut microbiota can profoundly modulate the efficacy of ICB therapies such as anti-PD-1/PD-L1 and anti-CTLA-4 antibodies. Only a subset of cancer patients derives durable benefit from ICB, and differences in commensal microbiota have emerged as one explanation for this variability: patients who respond to ICB tend to harbor distinct gut microbial communities compared to non-responders​ [3]. Further fecal microbiota transplantation (FMT) experiments have demonstrated causality: germ-free or antibiotic-treated mice receiving stool from ICB-responsive patients show improved tumor control under ICB, whereas those colonized with stool from non-responders fail to respond​. These findings establish the microbiome as a key determinant of ICB success across multiple cancer types, including melanoma, NSCLC, RCC, and others​ [15, 16, 242–247].

Specific gut bacteria have been identified that enhance anti-tumor immunity and improve ICB outcomes. In melanoma patients treated with anti-PD-1, responders were found to have significantly higher microbial diversity and a greater abundance of Ruminococcaceae bacteria in their guts than non-responders​ [192]. Another study of metastatic melanoma reported that *B. longum*, *Collinsella aerofaciens*, and *Enterococcus faecium* were highly enriched in responders, and when germ-free mice were reconstituted with feces from these responding patients, the mice showed improved tumor control, heightened T-cell activity, and increased efficacy of anti-PD-L1 therapy [193]. Consistent observations have been made in other cancers as well: for example, in NSCLC and RCC patients on anti-PD-1, clinical responders often harbor *Akkermansia muciniphila* and other “beneficial” anaerobes that are depleted in non-responders​ [176].

Beneficial microbes appear to prime the host immune system for a robust anti-tumor response. In a melanoma mouse model, feeding mice *Bifidobacterium* improved tumor control to the same degree as anti-PD-L1 therapy, and combining *Bifidobacterium* with anti-PD-L1 nearly eradicated the tumors​ [174]. This microbiota-driven effect was linked to enhanced tumor-specific T-cell responses: *Bifidobacterium*-treated mice showed increased IFN-γ production and CD8^+^ T-cell accumulation in tumors​ [174]. Similarly, *Akkermansia muciniphila* has been shown to reinvigorate anti-PD-1 responses after dysbiosis: in antibiotic-treated cancer-bearing mice, supplementation with *A. muciniphila* restored responsiveness to PD-1 blockade in an interleukin-12-dependent manner, increasing the recruitment of CCR9^+^CXCR3^+^CD4^+^ T lymphocytes into the TME [176]. For CTLA-4 blockade, *B. fragilis* can induce Th1-weighted systemic immunity, as shown in germ-free or antibiotic-treated mice, anti-CTLA-4 therapy had no anti-tumor effect until *B. fragilis* bolstering Il-12 production was introduced [248].

As certain microbes can augment ICB, an unfavorable gut microbiome can dampen immunotherapy efficacy. Patients with low gut microbial diversity or dysbiotic communities tend to respond poorly to checkpoint blockade​ [176, 192]. Compared to PD-1 responders with abundant Ruminococcaceae bacteria, non-responders’ microbiomes were often dominated by bacteria associated with a less immunostimulatory profile (such as Bacteroidales), and these patients exhibited impaired systemic and intratumoral T-cell activity [192]​. Broad-spectrum antibiotic use leading to dysbiosis is a clear negative influence: cancer patients who received antibiotics around the start of ICB treatment have significantly reduced response rates and poorer survival on anti-PD-1 therapy​ [176].

Various gut microbiome characteristics, including community structures, taxonomic compositions, and molecular functions, have emerged as key biomarkers for predicting immunotherapy responses and immune-related adverse events, with several clinical observations reinforcing the microbiota-ICB connection [249]. Two recent clinical trials in melanoma provided the proof-of-concept that FMT can induce ICB responses: a subset of PD-1-refractory melanoma patients achieved tumor regression or disease stabilization after receiving FMT from a donor who had responded to anti-PD-1 [250, 251]. Another phase I clinical trial demonstrates that, in patients with advanced melanoma, the response rate to combined FMT and anti-PD-1 therapy increases to 65% when an FMT is administered before the first anti-PD-1 dose [18]. These pioneering trials illustrate the therapeutic potential of modulating the microbiome and bolster the idea of using baseline microbiome composition as a predictive biomarker for immunotherapy.

### Chimeric antigen receptor (CAR)-T cell therapy

The gut microbiota has emerged as a key modulator of various cancer therapies, including immunotherapy. While its role in immune checkpoint blockade has been extensively studied, its influence on CAR-T cell therapy is a growing area of interest. Preliminary clinical evidence suggests that gut microbiota composition may affect both the response to and toxicity of CAR-T cell therapy, highlighting its potential as a modifiable factor in treatment outcomes.

Baseline microbial composition appears to correlate with the efficacy of CAR T-cell therapy, suggesting that specific bacterial populations present before infusion may influence therapeutic success [252]. A study analyzing patients with acute lymphoblastic leukemia (ALL) and non-Hodgkin lymphoma (NHL) treated with CD19 CAR T cells found that a high abundance of obligate anaerobes such as *Ruminococcus, Bacteroides, Faecalibacterium,* and *Akkermansia* was associated with a complete response at day 100 [253]. This pattern mirrors findings in immune checkpoint blockade, where similar microbial profiles are linked to improved responses [254]. Conversely, exposure to broad-spectrum anaerobe-targeting antibiotics, such as piperacillin-tazobactam, imipenem-cilastatin, and meropenem (PIM), within four weeks before CAR T-cell infusion was associated with decreased overall survival and reduced alpha diversity of the gut microbiome [252, 253, 255]. This suggests that antibiotic-induced microbiota disruption may negatively impact CAR T-cell therapy outcomes.

Additional studies have supported these observations. In patients with NHL, a higher abundance of *Bifidobacterium longum* and increased peptidoglycan biosynthesis correlated with long-term survival and therapeutic response, independent of other clinical factors [256]. Further, *Lachnospira pectinoschiza* and *Akkermansia muciniphila* were significantly associated with CD3⁺ and CD4⁺ T cell counts at the time of apheresis, suggesting that these bacteria may influence the quality of T cells collected for CAR T-cell manufacturing [257]. Notably, *Akkermansia* was also linked to enhanced CAR T-cell product quality and function [257]. Interestingly, one study observed that patients exposed to oral vancomycin post-infusion exhibited a higher peak expansion of CAR-T cells, suggesting that specific antibiotic-mediated microbiota modifications could enhance expansion kinetics, though further investigation is needed [258].

Beyond efficacy, the gut microbiota may also influence treatment-associated toxicities, including immune effector cell-associated neurotoxicity syndrome (ICANS) and cytokine release syndrome (CRS). Pre-infusion exposure to PIM antibiotics was linked to an increased incidence of ICANS, particularly in NHL patients, although no statistically significant association with CRS was observed in the combined NHL and ALL cohort [253]. A separate study investigating microbiome changes during CAR T-cell therapy in patients with B-cell malignancies found that while microbial composition was associated with treatment response, its direct impact on CRS severity remained unclear. However, in myeloma patients, distinct CRS grades were associated with differential abundance of amino acid metabolism pathways, suggesting a potential link between microbial metabolism and cytokine-driven toxicities [259].

Further evidence indicates that microbiota composition can modulate CAR T-cell therapy-related CRS and overall therapeutic response in patients with hematologic malignancies. Differences in microbial composition may lead to variable clinical outcomes by influencing host immunity and cytokine release. For example, amino acid metabolism by gut microbes has been implicated in shaping both innate and adaptive immune responses, potentially contributing to variations in CRS severity [259]. This raises the possibility that microbiota modulation could serve as a therapeutic strategy to attenuate CAR T-cell-associated toxicities, though specific mechanisms and effective interventions remain under investigation [260].

In conclusion, the gut microbiota plays a significant role in shaping the efficacy and toxicity of CAR T-cell therapy. Specific bacterial taxa and overall microbial diversity appear to be associated with therapeutic outcomes, and antibiotic-induced dysbiosis may negatively impact treatment success [261, 262]. Further research is needed to fully elucidate the complex interplay between the microbiota and CAR T-cell therapy, potentially paving the way for microbiome-based strategies to enhance efficacy and mitigate adverse events such as CRS.

### Allo-HSCT

The gut microbiota plays a crucial role in determining the success of allogeneic hematopoietic stem cell transplantation (allo-HSCT), significantly influencing the risk of graft-versus-host disease (GVHD), treatment-related mortality, and overall survival. Dysbiosis has been consistently associated with poor post-transplant outcomes [263]. Patients who experience a significant decline in gut microbial diversity during HSCT exhibit higher mortality rates, underscoring the importance of a balanced microbiota in transplantation success [263]. Specific microbial alterations also contribute to transplant outcomes. For instance, an overrepresentation of pro-inflammatory bacteria such as *Enterococcus* has been linked to severe GVHD and worse survival rates [264, 265]. In contrast, the presence of beneficial commensals—including members of the *Ruminococcaceae* and *Lachnospiraceae* families—correlates with reduced GVHD severity and improved survival [266]. Notably, *Eubacterium limosum* dominance has been associated with a lower risk of disease progression and relapse, while the abundance of *Blautia* has been linked to reduced GVHD-related mortality [264, 267].

Antibiotic prophylaxis, a common component of HSCT protocols, further disrupts the gut microbiota, exacerbating dysbiosis and increasing post-transplant complications [263]. While broad-spectrum antibiotics can deplete beneficial microbial populations and heighten GVHD risk, certain antibiotics, such as rifaximin, have demonstrated protective effects by preserving microbiota diversity and improving overall survival [263, 265, 268, 269]. Given these findings, strategies to maintain gut microbial diversity may be critical for optimizing allo-HSCT outcomes.

Microbiota-targeted interventions, such as FMT, are emerging as promising strategies to enhance post-transplant recovery [263, 265, 266, 270]. FMT has shown efficacy in treating steroid-refractory acute GVHD and recurrent *Clostridioides difficile* infections in HSCT recipients [265, 270]. In addition, the restoration of microbial diversity through FMT has been linked to clinical improvements in GVHD patients, reinforcing the potential of microbiota modulation as a therapeutic approach [270]. Therefore, gut microbiota composition may serve as a predictive biomarker for allo-HSCT success and a target for interventions aimed at improving transplant efficacy and patient survival.

### Other immunotherapies

Recent advances in immuno-oncology have illuminated the complex interplay between host immunity and the gut microbiome, revealing that commensal bacteria critically shape the tumor-immune landscape. Mounting evidence suggests that microbiota composition can modulate both innate and adaptive immune responses, profoundly impacting the efficacy of cancer immunotherapies—including cancer vaccines, T cell receptor (TCR)-based therapies, and oncolytic viruses.

Dendritic cells (DCs) serve as key antigen-presenting cells that bridge innate and adaptive immunity. Their activation and function are highly sensitive to microbial signals. Specific gut-resident bacteria have been shown to influence DC maturation and cytokine production, thereby regulating the priming and expansion of tumor-specific T cells. These findings suggest that microbiome-derived cues could determine the magnitude and quality of antitumor responses elicited by cancer vaccines. By modulating antigen presentation and the immunogenicity of tumor-associated antigens, the gut microbiota emerges as a pivotal factor shaping vaccine responsiveness [271, 272].

The efficacy of TCR-engineered T cell therapies hinges on optimal T cell priming, trafficking, and persistence. Studies in germ-free or antibiotic-treated murine models have revealed that the absence of commensal microbes impairs antigen-specific CD8⁺ T cell responses and reduces the therapeutic benefit of adoptive cell transfer [273]. Mechanistically, microbial-associated molecular patterns and metabolites—including short-chain fatty acids (SCFAs) such as butyrate and propionate—have been shown to enhance memory T cell generation and sustain effector functions within the tumor microenvironment [274, 275]. Notably, the presence of taxa such as *Bifidobacterium* and *Akkermansia muciniphila* correlates with favorable responses to immune checkpoint inhibitors, raising the possibility that similar microbial consortia may bolster TCR-based immunotherapies. Additionally, microbial translocation into tumors has been implicated as a modulator of the intratumoral T cell landscape, potentially reprogramming the functional phenotypes of tumor-infiltrating lymphocytes (TILs) [276]. These insights open avenues for microbiome-targeted interventions—including fecal microbiota transplantation, dietary modulation, and administration of defined bacterial consortia—as adjuvants to improve TCR therapeutic outcomes.

The immunogenicity of oncolytic virus (OV) therapy is also influenced by the gut microbiome. In preclinical melanoma models, beneficial bacteria such as *Bifidobacterium* have been shown to amplify the antitumor activity of oncolytic adenoviruses by increasing tumor infiltration of CD8⁺ T cells while depleting regulatory T cells (Tregs), which dampen immune responses [277, 278]. Similarly, in colorectal cancer models, manipulation of the intestinal microbiota—via probiotics or fecal microbiota transplantation—enhances OV efficacy through a shift toward immune activation. These data support the concept that gut microbial composition is a critical determinant of OV-mediated immune priming and tumor regression, and suggest that microbiome-based strategies could serve as valuable adjuncts to viral immunotherapies.

Together, these findings underscore a unifying theme: the gut microbiome is a potent regulator of cancer immunotherapy outcomes across diverse platforms. Integration of microbiome science with immunotherapeutic design may unlock novel synergies, paving the way for personalized and more effective anticancer interventions.

## Clinical applications

Harnessing the microbiome to improve cancer immunotherapy has rapidly moved from correlative studies into clinical exploration. Researchers and clinicians are testing several strategies to modulate a patient’s microbiome with the goal of enhancing anti-tumor immune responses or managing immunotherapy-related toxicities. Each strategy has shown promise in clinical trials or case studies, across various cancer types, as outlined below.

### FMT

FMT has gained the most attention as a method to globally reset the gut microbiota and potentially overcome tumor resistance to immunotherapy​ [279]. Early proof-of-concept trials have shown that FMT from cancer patients who responded well to ICB was given to patients who had failed prior immunotherapy, with encouraging results in advanced melanoma [250, 251, 280]. Most studies use stool from donors who had remarkable immunotherapy responses (to maximize the likelihood of transferring beneficial microbes), while others use healthy donors selected for harboring microbiota profiles similar to responders​ [281]. For example, an ongoing Phase II trial (NCT03341143) in refractory melanoma is testing FMT plus pembrolizumab using responder donors​. In addition, a phase I trial in ICI-naïve melanoma patients investigated FMT from healthy donors given orally one week before anti-PD-1 therapy (NCT03772899). The combination of FMT and anti-PD-1 yielded a 65% objective response rate, including four complete responses, surpassing the outcomes typically seen with immunotherapy alone. Trials are now underway in other cancers, such as RCC, CRC, and NSCLC, to evaluate FMT’s potential to boost checkpoint inhibitors [282]​.

Beyond enhancing efficacy, FMT is also being explored as a therapy for managing immunotherapy-induced toxicities. Clinical data have shown that FMT from healthy donors can ameliorate immune-related colitis related to ICB: four out of five patients with refractory ICB-associated colitis experienced rapid improvement in symptoms after FMT, and two patients who later relapsed responded successfully to a second FMT [283]. These outcomes underscore that restoring a healthy microbial balance can reduce inflammatory toxicity while potentially permitting patients to continue immunotherapy.

### Probiotics and live biotherapeutic products

Another approach to modulate the microbiome in cancer patients is the use of probiotics or defined bacterial consortia. Off-the-shelf probiotics like *Lactobacillus* or *Bifidobacterium* supplements are widely available, but their unregulated status and variable quality have raised concerns in the oncology setting​ [281, 284]. In fact, uncontrolled use of commercial probiotics is not generally recommended during immunotherapy​ [281]. The reason is that the impact of generic probiotics on the complex gut ecosystem and immune response is still poorly understood, and some correlative studies even suggest potential negative effects. For instance, a study in melanoma patients found that those who self-reported taking over-the-counter probiotics had a lower gut microbiome diversity and a trend toward worse ICB treatment outcomes​ [285].

On the other hand, clinically selected probiotic therapies are showing promise when targeted appropriately. One notable example is a live biotherapeutic product containing *Clostridium butyricum* (strain CBM588). In a randomized phase I trial in metastatic RCC (NCT03829111), patients received combination of nivolumab and ipilimumab with or without oral CBM588 supplementation [281]. The addition of CBM588 led to markedly improved outcomes: the objective response rate was 58% with CBM588 compared to 20% in the control group, and median progression-free survival increased from 2.5 months to 12.7 months [286]. No unexpected toxicities were seen with the probiotic; in fact, the safety profile was similar between the two groups [286]. Several other probiotic-based immunotherapy adjuvants are under investigation. For instance, an oral product containing *B. animalis subsp. lactis* (EDP1503) has been tested in a phase I trial (NCT03775850) with ICB for treating advanced metastatic CRC, triple-negative breast cancer, and checkpoint inhibitor relapsed tumors. Additionally, consortia of multiple commensal strains are being developed to reconstitute a favorable microbiome more comprehensively. One consortium, VE800, which consists of 11 strains of commensal bacteria enriched in responders, has been evaluated in combination with nivolumab in patients with advanced microsatellite-stable CRC and other difficult-to-treat tumors (NCT04208958)​ [287]. Nonetheless, a key challenge for probiotic approaches is ensuring that the administered microbes can engraft and persist in the patient’s gut. Achieving this may require techniques such as antibiotic pre-conditioning or the use of biofilm-enhancing adjuncts [281]. For example, using smectite to establish colonization sites specifically for probiotics has been shown to enhance the efficacy of immunotherapy in mice [288]. Overall, while still experimental, probiotic and live biotherapeutic strategies are emerging as a practical alternative to FMT, aiming to deliver specific immune-potentiating microbes in a controlled manner.

### Dietary interventions and prebiotics

Diet is a powerful determinant of the gut microbiota and represents a non-invasive strategy to modulate microbial composition and metabolism. In particular, diets rich in fiber and other prebiotics can promote the expansion of short-chain fatty acid-producing and anti-inflammatory bacteria, which in turn may improve anti-tumor immunity​ [192, 193, 289, 290]. Clinical observations have already linked diet to immunotherapy outcomes. A retrospective study of 128 melanoma patients on PD-1 inhibitors found that those with sufficient dietary fiber intake had a longer median progression-free survival, and they tended to respond better to treatment​ [285]. The longest disease control was seen in patients who had high fiber consumption without probiotic use, correlating with greater gut microbiome diversity though this subgroup difference did not reach statistical significance​ [281, 285].

Prospective trials are now testing dietary modulation as an adjunct to immunotherapy. For example, the ongoing Diet and Immune Effects Trial (DIET) study is a randomized, controlled trial of a high-fiber diet intervention versus a healthy control diet in melanoma patients receiving ICB. In this phase II trial, participants are placed on a strictly controlled diet high in diverse fibers to see if it improves response rates or survival compared to a standard diet, and early feasibility work showed that such dietary interventions are well-tolerated with compliance​ [291]. Besides fiber, other dietary components are being investigated: an analysis found melanoma patients who responded to ICB had significantly higher omega-3 fatty acid intake than non-responders [292]​. Prebiotic supplements like inulin can selectively stimulate fiber-fermenting gut microbes, such as *Faecalibacterium* and *Bifidobacterium* species, and in mouse models, adding an inulin-based prebiotic enhanced anti-PD-1 therapy, increasing the abundance of essential commensal microbiome along with their SCFA metabolites [289, 290, 293]​. These findings suggest that specific diets or fiber supplements might mimic the effects of a “beneficial” microbiome by nourishing immunologically favorable bacteria.

### Antibiotics and microbiome management

Given the microbiome’s importance in ICI efficacy, there is strong interest in avoiding practices that unintentionally harm the microbiome during cancer therapy. Antibiotics can cause a loss of gut microbial diversity and wipe out key commensals, potentially blunting the patient’s immune response to tumors​ [294]. A recent meta-analysis including 12,493 patients across 13 types of cancer found that concomitant antibiotic use was associated with a nearly two-fold higher risk of disease progression and death under immunotherapy [294]​. The timing and spectrum of antibiotics also mattered during the treatment—the negative effect was the strongest when broad-spectrum antibiotics were given in the window from 60 days before through 30 days after starting ICI therapy [294]​. These data underscore the importance of antibiotic management in cancer patients.

Interestingly, not all antibiotics have identical effects on the microbiome, and there are experimental efforts to leverage selective antimicrobial treatments to shape gut flora in favorable ways. For example, in preclinical studies, treating mice with vancomycin can improve anti-CTLA-4 (ipilimumab) therapy efficacy​ [248, 295]. However, such strategies remain unproven in humans. The clinical case of treating recurrent colitis with repeating FMT after antibiotic-induced dysbiosis provides a proof of principle that microbiota repair after antibiotics can restore immune equilibrium​ [283]. Overall, the clinical guidance is to use antibiotics cautiously in cancer immunotherapy patients, balancing infection control with the recognition that the gut microbiome is an important factor in treatment success​ [294].

## Mitochondria-microbiome crosstalk in tumor immunosurveillance

Mitochondria and the microbiome form a bidirectional metabolic-immunologic axis that influences cancer risk and therapy responses. On the host to microbe axis, mitochondrial metabolic reprogramming alters the production and routing of metabolites, such as succinate, fumarate, citrate/acetyl-CoA, lactate, β-hydroxybutyrate, and itaconate that condition epithelial and myeloid programs. These shifts remodel barrier function, oxygen tension, luminal pH, and antimicrobial defenses, thereby selecting for specific microbial guilds. For example, succinate signaling through SUCNR1 drives inflammatory remodeling with consequences for community structure, whereas IRG1-derived itaconate restrains excessive inflammation and imposes antimicrobial pressure that feeds back on microbial ecology [296–298].

On the opposite axis, bacterial metabolites reprogram mitochondrial metabolism and fate decisions in immune cells. SCFAs augment acetyl-CoA pools and bias T cells toward FAO/OXPHOS-supported states, calibrating effector, regulatory, and memory programs [190, 299–303]. Tryptophan catabolites engage the aryl hydrocarbon receptor (AhR) to reshape mitochondrial activity and transcriptional profiles. Microbial bile-acid derivatives tune the hepatic CXCL16-CXCR6 circuit, promoting the recruitment and function of CXCR6⁺ NKT cells and, in turn, shaping liver-selective tumor immunosurveillance [86, 304].

Succinate, a TCA intermediate that accumulates in hypoxia/inflammation, activates the SUCNR1 receptor on stromal and immune cells, promoting inflammatory rewiring and shaping anti-tumor immunity. SUCNR1 also shows cancer- and subtype-specific associations and may intersect with microbiome patterns. Therapeutically, SUCNR1 modulators and strategies that normalize succinate flux are emerging [305–307]. Lactate, which is abundant in many TMEs, reprograms myeloid and T-cell mitochondria, skews effector function, and may influence microbe nutrient niches; inhibitors of lactate transport/signaling are in development [308]. Moreover, itaconate, generated by IRG1 in mitochondria of activated myeloid cells, exerts antimicrobial pressure, dampens exuberant inflammation via KEAP1/NRF2 signaling, and can indirectly reshape tumor immunity [309–311].

## Future directions

The rapid expansion of microbiome research has introduced promising approaches for advancing cancer diagnostics and treatment. As evidence continues to grow regarding the role of microbiome in tumor development, immune modulation, and therapeutic response, new clinical applications are beginning to emerge. The future of oncology-focused microbiome research will likely depend on technological advancements in profiling methods, the development of microbiome-targeted therapies, and addressing key translational barriers.

Recent improvements in sequencing technologies and bioinformatics have enhanced our ability to characterize cancer-associated microbiomes with greater precision. Techniques such as 16S rRNA gene sequencing and metagenomic shotgun sequencing enable detailed taxonomic and functional profiling of microbial communities specifically. In addition, multi-omic approaches, including metatranscriptomics, metabolomics, and proteomics, are offering more comprehensive insights into the dynamic interactions between host and microbiota. When integrated with clinical and immunological data using systems biology frameworks and machine learning, these datasets hold potential for the development of microbiome-based biomarkers for cancer diagnosis, prognosis, and therapy prediction [292, 312].

Several promising strategies are emerging in the field of microbiota-targeted therapies. One such strategy involves modulating the host immune response through the microbiome. Fecal microbiota transplantation (FMT), which has shown success in treating recurrent *Clostridioides difficile* infections, is currently being investigated in cancer patients to help restore a favorable microbiome composition and improve responses to immune checkpoint inhibitors (ICIs). Early-phase clinical trials have shown that FMT from ICI-responsive donors may help restore anti-tumor immunity in patients with advanced melanoma who are refractory to immunotherapy [250, 251]. In parallel, the use of defined probiotic consortia—colonization with specific beneficial microbes—has been associated with improved responses to ICIs and reduced treatment-related toxicity [313]. Another innovative approach involves engineered microbial therapeutics: genetically modified bacteria designed to deliver therapeutic molecules or modulate the immune environment directly within tumors. Preclinical models have demonstrated that these engineered microbes can selectively localize to tumors and release cytokines or immune modulators within the tumor microenvironment [314].

Despite these advances, several challenges remain before microbiome-based therapies can be widely implemented in clinical oncology. A key obstacle is the significant inter-individual variability in microbiome composition, which is influenced by factors such as diet, geography, medication use, and host genetics. This variability complicates the standardization of therapeutic strategies [315]. Moreover, regulatory and safety concerns persist, particularly regarding classification, manufacturing, and quality control of FMT and engineered probiotics. Ensuring long-term safety and avoiding unintended immune responses are essential. Lastly, incorporating microbiome analysis into routine clinical practice will require the development of affordable, rapid, and reliable diagnostic tools to enable widespread adoption [316]. Continued interdisciplinary research, along with collaboration between microbiologists, oncologists, immunologists, and regulatory bodies, will be critical to translating microbiome science into safe and effective cancer therapies.

## Conclusion

The cumulative evidence presented in this review underscores that the commensal microbiota is a pivotal, context-dependent regulator of both tumorigenesis and anticancer immune responses. In some settings, dysbiosis promotes tumor initiation and progression by driving chronic inflammation, reprogramming metabolic signaling, and subverting immune surveillance. Conversely, in other contexts a balanced microbiome can actively suppress malignancy, strengthening epithelial barriers, producing anti-inflammatory metabolites, and bolstering immune-mediated tumor surveillance. These influences highlight the microbiome’s dual role in cancer progression, functioning as both an instigator and a protector. As a result, the microbiome has emerged as a critical extrinsic factor that not only shapes cancer risk but also impacts how established tumors behave and respond to therapy.

Equally significant is the microbiome’s influence on therapeutic outcomes, particularly in immunotherapy. Patient studies have shown that commensal microbial profiles can modulate the efficacy of immune checkpoint inhibitors, CAR-T cell therapies, and other treatments with certain gut bacteria linked to improved responses and fewer immune-related toxicities, whereas antibiotic-induced dysbiosis or loss of microbial diversity can undermine treatment success. These insights have catalyzed the exploration of microbiome-targeted interventions as novel adjuncts to standard oncology care. Early clinical trials offer proof-of-concept that manipulating a patient’s microbiome can enhance anticancer immunity or even restore responsiveness to treatment. For instance, FMT from immunotherapy responders has restored ICB efficacy in patients with refractory melanoma. Similarly, high-fiber diets and probiotics have been associated with more favorable immunotherapy outcomes. While such approaches are still under studied, they illustrate the exciting clinical potential of employing the microbiome to improve patient outcomes.

Realizing the full therapeutic potential of microbiome-targeted interventions will require overcoming several significant challenges. The complexity and variability of microbiome across individuals complicate efforts to establish universally effective microbial treatments. Moreover, the dynamic interplay between the immune system and microbiome presents further hurdles in predicting treatment outcomes and designing effective interventions. Thus, a deeper mechanistic understanding of host-microbiome interactions in cancer will be essential. Integrating personalized microbiome profiling into precision oncology could enable clinicians to tailor microbial interventions to individual patients, maximizing efficacy while minimizing risks. By harnessing the patient’s microbiome alongside conventional treatments, future cancer therapies may achieve more durable remissions and reduced toxicity, ultimately transforming our approach to cancer prevention and treatment.

## Data Availability

No datasets were generated or analyzed during the current study.

## Abbreviations

SCFAs: Short-chain fatty acids
TNBC: Triple-negative breast cancer
CRC: Colorectal cancer
TLR: Toll-like receptors
AOM/DSS: Azoxymethane/dextran sodium sulfate
ccRCC: Clear cell renal cell carcinoma
HCC: Hepatocellular carcinoma
GM: Gut microbiome
MAMPs: Microbe-associated molecular patterns
CLDs: Chronic liver diseases
NAFLD: Non-alcoholic fatty liver disease
PAMPs: Pathogen-associated molecular patterns
LPS: Lipopolysaccharide
ROS: Reactive oxygen species
Bas: Bile acids
DCA: Deoxycholic acid
isoallo-LCA: Isoallolithocholic acid
CagA: Cytotoxin-associated gene A
cag PAI: Cag pathogenicity island
OMPs: Outer membrane proteins
DCs: Dendritic cells
NK: Natural killer
NSCLC: Non-small cell lung cancer
Tregs: Regulatory T cells
TME: Tumor microenvironment
FMT: Fecal microbiota transplantation
GF: Germ-free
ICIs: Immune checkpoint inhibitors
ID2: Inhibit DNA binding protein 2
IgA: Immunoglobulin A
CSR: Class-switch recombination
MET4: Microbial ecosystem therapeutic 4
ICB: Immune checkpoint blockade
RCC: Renal cell carcinoma
CAR: Chimeric antigen receptor
ALL: Acute lymphoblastic leukemia
NHL: Non-Hodgkin lymphoma
PIM: Piperacillin-tazobactam, imipenem-cilastatin, and meropenem
ICANS: Immune effector cell-associated neurotoxicity syndrome
CRS: Cytokine release syndrome
allo-HSCT: Allogeneic hematopoietic stem cell transplantation
GVHD: Graft-versus-host disease
TCR: T cell receptor
TILs: Tumor-infiltrating lymphocytes
OV: Oncolytic virus
DIET: Diet and immune effects trial
